# Keypress-Based Musical Preference Is Both Individual and Lawful

**DOI:** 10.3389/fnins.2017.00136

**Published:** 2017-05-02

**Authors:** Sherri L. Livengood, John P. Sheppard, Byoung W. Kim, Edward C. Malthouse, Janet E. Bourne, Anne E. Barlow, Myung J. Lee, Veronica Marin, Kailyn P. O'Connor, John G. Csernansky, Martin P. Block, Anne J. Blood, Hans C. Breiter

**Affiliations:** ^1^Warren Wright Adolescent Center, Department of Psychiatry and Behavioral Sciences, Feinberg School of Medicine, Northwestern UniversityChicago, IL, USA; ^2^Applied Neuromarketing Consortium, Medill, Kellogg, and Feinberg Schools, Northwestern UniversityEvanston, IL, USA; ^3^David Geffen School of Medicine, University of California, Los AngelesLos Angeles, CA, USA; ^4^Northwestern University and Massachusetts General Hospital Phenotype Genotype Project in Addiction and Mood DisordersBoston, MA, USA; ^5^Medill Integrated Marketing Communications, Northwestern UniversityEvanston, IL, USA; ^6^Music Department, Bates CollegeLewiston, ME, USA; ^7^KV 265, The Communication of Science through ArtWillow Springs, IL, USA; ^8^Department of Psychiatry and Behavioral Sciences, Feinberg School of Medicine, Northwestern UniversityChicago, IL, USA; ^9^Mood and Motor Control Laboratory, Department of Psychiatry, Massachusetts General HospitalBoston, MA, USA; ^10^Laboratory of Neuroimaging and Genetics, Department of Psychiatry, Massachusetts General HospitalBoston, MA, USA; ^11^Department of Neurology, Massachusetts General HospitalBoston, MA, USA

**Keywords:** relative preference theory, music, reward, preference, approach, avoidance

## Abstract

Musical preference is highly individualized and is an area of active study to develop methods for its quantification. Recently, preference-based behavior, associated with activity in brain reward circuitry, has been shown to follow lawful, quantifiable patterns, despite broad variation across individuals. These patterns, observed using a keypress paradigm with visual stimuli, form the basis for relative preference theory (RPT). Here, we sought to determine if such patterns extend to non-visual domains (i.e., audition) and dynamic stimuli, potentially providing a method to supplement psychometric, physiological, and neuroimaging approaches to preference quantification. For this study, we adapted our keypress paradigm to two sets of stimuli consisting of seventeenth to twenty-first century western art music (Classical) and twentieth to twenty-first century jazz and popular music (Popular). We studied a pilot sample and then a separate primary experimental sample with this paradigm, and used iterative mathematical modeling to determine if RPT relationships were observed with high *R*^2^ fits. We further assessed the extent of heterogeneity in the rank ordering of keypress-based responses across subjects. As expected, individual rank orderings of preferences were quite heterogeneous, yet we observed mathematical patterns fitting these data similar to those observed previously with visual stimuli. These patterns in music preference were recurrent across two cohorts and two stimulus sets, and scaled between individual and group data, adhering to the requirements for lawfulness. Our findings suggest a general neuroscience framework that predicts human approach/avoidance behavior, while also allowing for individual differences and the broad diversity of human choices; the resulting framework may offer novel approaches to advancing music neuroscience, or its applications to medicine and recommendation systems.

## Introduction

Preference can be defined as the variable extent an individual will approach or avoid events and objects in the world based on their rewarding or aversive features (Lewin et al., 1935; Schneirla, 1959; Warren, 1963). Research on preference has emphasized the subjective nature of preferences (Kable and Glimcher, 2007; Lau and Glimcher, 2008). In music, individual preferences are thought to be quite heterogeneous, leading to use of individualized stimuli for neuroimaging and the concordant challenge of generalizing preference toward music across individuals (e.g., Blood and Zatorre, 2001; Osuch et al., 2009; Pereira et al., 2011; Trost et al., 2012; Salimpoor et al., 2013). Despite individual variation in preference, there appears to be common circuitry for emotional processing of music (e.g., Blood and Zatorre, 2001; Osuch et al., 2009; Pereira et al., 2011; Trost et al., 2012; Salimpoor et al., 2013), suggesting there may be biologically-based functions describing a general music response, which act like a scaffold upon which individual variation occurs.

Recently, preference-based behavioral variables measuring the intensity and patterns of keypressing to approach or avoid visual stimuli were shown to exhibit lawful relationships in humans (Breiter and Kim, 2008; Kim et al., 2010; Lee et al., 2015; Viswanathan et al., 2017). These behavioral variables were further associated with activation in brain reward circuitry by model-based functional MRI (Perlis et al., 2008; Gasic et al., 2009; Viswanathan et al., 2015). Lawfulness is a physics-based term describing patterns in data that are mathematically “discrete,” “recurrent,” “robust,” and “scalable” (Feynman, 1965; Mitra and Bokil, 2008; Kim et al., 2010). “Discreteness” refers to patterns having specific mathematical descriptions, while “recurrence” indicates a pattern repeats consistently across different stimuli and experiments. “Robustness” indicates that pattern cannot be generated from nor easily perturbed by noise, and “scalability” indicates that a pattern occurs at different levels of organization, such as from an individual pattern to a group level pattern (Sutton and Breiter, 1994; Breiter et al., 2006; Braeutigam, 2017). Identifying lawful patterns in behavioral data helps define the specifications that biology must fulfill (i.e., like the specs of a car built in a factory). Such specifications facilitate meaningful interpretation of biological measures underlying behavior (e.g., brain function).

Lawful equations characterizing preference with a keypress task were initially identified using visual stimuli (Breiter and Kim, 2008; Kim et al., 2010; Lee et al., 2015). The keypress task was developed out of a operant framework where each keypress action had an incremental consequence on stimulus view time (Aharon et al., 2001; Lee et al., 2015), and has been well-validated across multiple studies (Aharon et al., 2001; Elman et al., 2005; Strauss et al., 2005; Levy et al., 2008; Makris et al., 2008; Perlis et al., 2008; Gasic et al., 2009; Yamamoto et al., 2009; Kim et al., 2010; Lee et al., 2015; Viswanathan et al., 2015, 2017). It follows an intrinsic motivation framework devoid of external rewards, such as food or money (Deci and Ryan, 1985; Bandura, 1997), and quantifies reward/aversion by how much subjects approach or avoid stimuli—namely, to what extent subjects actively keypress to increase or decrease the amount of time they are exposed to predetermined categories of stimuli. The keypress task is a variant of techniques used to study effort-based decision-making (Walton et al., 2002, 2003, 2006). The task allows computation of metrics that quantify the magnitude and the predictability of participants' keypress-based preference behavior. These metrics include the mean number of keypresses subjects make to either approach (*K*^+^) or avoid (*K*^−^) stimuli within each category. Other metrics include the variance to approach (σ^+^) or avoid (σ^−^) stimuli, along with the Shannon entropy (i.e., information; see Shannon and Weaver, 1949) of the distribution of keypress counts to approach (*H*^+^) or avoid (*H*^−^) stimuli within each category. The Shannon entropy is a core variable in information theory that characterizes the degree of uncertainty across a set of responses (Shannon and Weaver, 1949). Collectively, these variables capture the decision-making about the valence of behavior (approach or avoidance) as well as judgments regarding its magnitude (intensity of keypressing) to describe relative preferences (Kim et al., 2010). We refer to this methodology and the lawful relationships it uncovers as *relative preference theory* (RPT).

**Figure 1:**
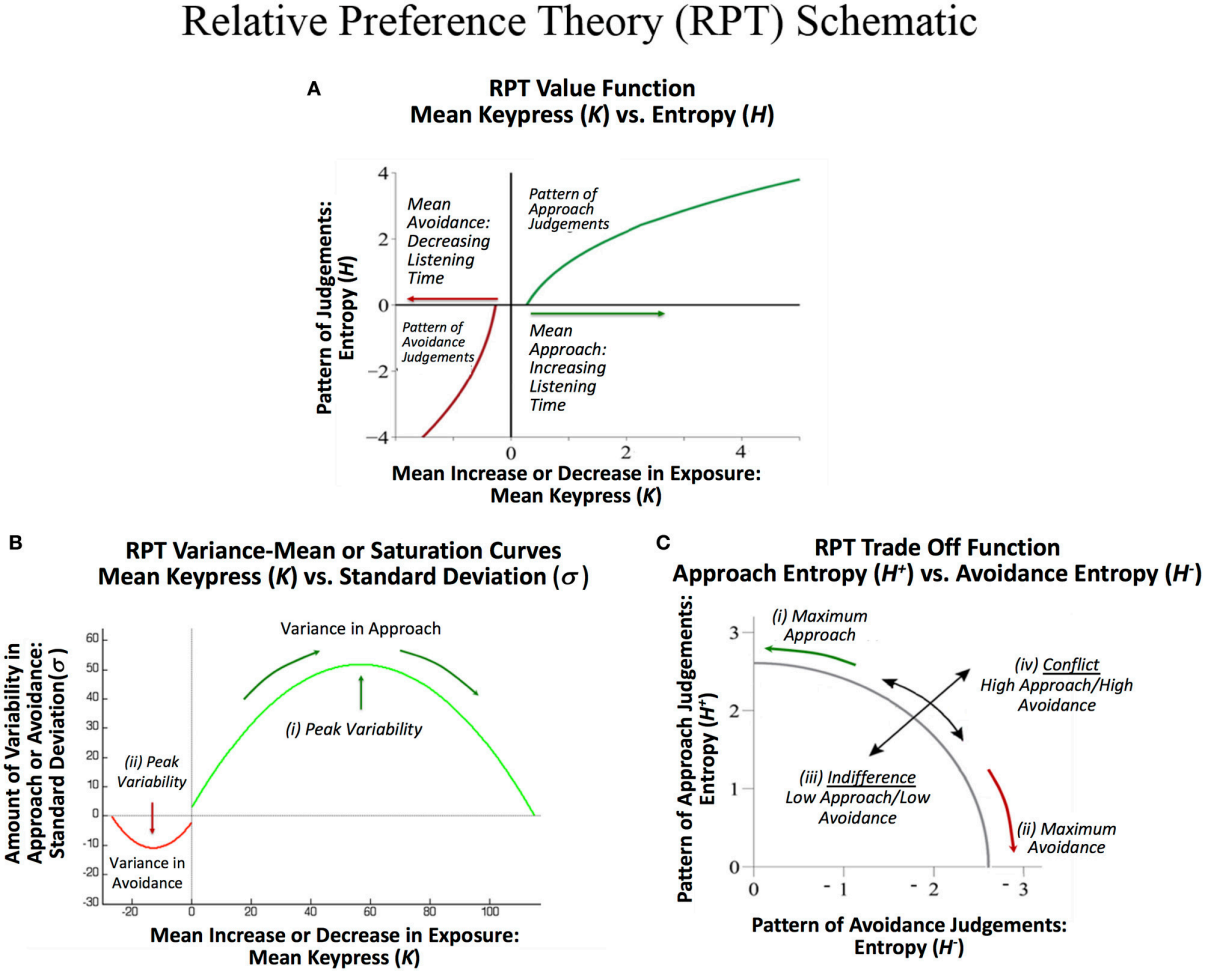
**Schematic of relative preference curves describing keypress responses to visual stimuli, and hypothesized to occur for music stimuli. (A)** A value function plotting the Shannon entropy (*H*^+^, *H*^−^) of the subject's keypress response patterns against the mean number of keypresses (*K*^+^, *K*^−^) to approach or avoidance the stimuli, computed separately for each music category. This function calibrates “wanting” against the pattern of previous judgments, and has a close resemblance to the value function in prospect theory. **(B)** A variance-mean relationship between the mean number of keypresses (*K*^+^, *K*^−^) plotted against the standard deviation of the keypress response patterns (σ^+^, σ^−^) for each category. This parabolic function describes limits to preference or its “saturation” by the intercepts on the x-axis. They further describe peaks in variability for approach (i) and for avoidance decisions (ii), respectively. These peaks represent a type of threshold beyond which an individual makes more approach and avoidance decisions as described under Markowitz's decision utility (dU = *K*− bσ, where *b* is a constant). **(C)** A trade off function between the approach entropy (*H*^+^) and avoidance entropy (*H*^−^). This function reflects a quantifiable trade-off between approach and avoidance decisions (i) and (ii), and also graphs the extent to which an individual has low approach and low avoidance simultaneously, resembling indifference (iii), or high approach and high avoidance simultaneously, resembling conflict (iv).

RPT is characterized in part by relationships between these three sets of behavioral variables {*K, H*, σ}. These relationships include: (1) A value function plotting the Shannon entropy (*H*^+^, *H*^−^), against the mean number of keypresses (*K*^+^, *K*^−^) for approach or avoidance toward a suite of objects. This function is referred to as a value function given it calibrates “wanting” against the pattern of previous judgments, and is consistent with the prospect theory value function discussed below. (2) A variance-mean relationship is observed between the mean number of keypress responses (*K*^+^, *K*^−^) plotted against the standard deviation of keypress responses (σ^+^, σ^−^). This relationship is characterized by increasing variance up to a peak followed by decreasing variance back to baseline. This function describes limits to preference or its “saturation.” (3) A trade-off function between the approach entropy (*H*^+^) and avoidance entropy (*H*^−^) was also identified, defining how bundles of approach decisions were balanced with bundles of avoidance decisions as a quantifiable trade-off between approach and avoidance. These relationships have been schematized in Figures 1A–C. Together, RPT provides a framework for calibrating the relative value of stimuli, with two types of control functions around value—namely, a limit to value, and a tradeoff between positive and negative value.

RPT relationships with {*K, H*, σ} have been connected to traditional reward and aversion circuitry, genetic polymorphisms, and neuroeconomic measures. The mean keypress response (*K*^+^, *K*^−^) has been associated with brain morphometry for paralimbic reward/aversion structures such as the insula that have strong connections with subcortical reward regions, and describe a significant abnormality between structure and behavior in addiction (Makris et al., 2008). It has further been associated with activity in brain regions such as the nucleus accumbens (NAc), amygdala, insula, and other reward regions, including when this activity in reward circuitry was associated with genetic polymorphisms related to *CREB1* and *BDNF* (Perlis et al., 2008; Gasic et al., 2009). The slopes of the (*K, H*) value function have also been directly connected to NAc activity (Viswanathan et al., 2015), consistent with other neuroeconomic studies linking the NAc and amygdala to prospect theory-based metrics (Tom et al., 2007; De Martino et al., 2010; Canessa et al., 2013). The RPT value function closely resembles that observed in prospect theory (Kahneman and Tversky, 1979; Figure 1A), showing a convex avoidance curve, a concave approach curve, and a steeper slope for the avoidance curve than the approach curve, consistent with the concept of “loss aversion” (Tversky and Kahneman, 1992), but adapted to a non-monetary framework (Lee et al., 2015). Prospect theory has also been associated with parametric activation in the same reward regions in humans as RPT (Breiter et al., 2001; Glimcher and Rustichini, 2004). The variance-mean relationship (*K*, σ) is reminiscent of Markowitz's quantification of decision utility (Markowitz, 1952; Kim et al., 2010; Figure 1B). Other neuroeconomic studies have directly connected Markowitz's decision utility to brain activation of reward systems in humans (D'Acremont et al., 2009; Mohr et al., 2010; Wunderlich et al., 2011).

The neuroimaging and neuroeconomics findings with RPT directly overlap findings in music neuroscience using psychometric, physiological, and neuroimaging methods. For example, fundamental work established the relationship between music-induced emotion and reward processing, specifically in the NAc and connected reward regions (e.g., Blood and Zatorre, 2001; Osuch et al., 2009; Pereira et al., 2011; Trost et al., 2012; Salimpoor et al., 2013). Current research continues to investigate the neural underpinnings of musical reward through examinations of stimulus characteristics (for reviews, see: Koelsch et al., 2006; Levitin and Tirovolas, 2009; Koelsch, 2010; Zatorre and Salimpoor, 2013), exposure (Schellenberg et al., 2008; Margulis et al., 2009; North and Hargreaves, 2010; Ladinig and Schellenberg, 2012), and personal attributes such as personality traits (Rentfrow and Gosling, 2003; Chamorro-Premuzic and Furnham, 2007; Rentfrow et al., 2011; Mas-Herrero et al., 2013; Martínez-Molina et al., 2016), media consumption (Chamorro-Premuzic and Furnham, 2007; Lamont and Webb, 2010; Mas-Herrero et al., 2013; Hollebeek et al., 2016; Martínez-Molina et al., 2016) and culture (Morrison et al., 2003, 2008; Wong et al., 2009, 2012; Berns et al., 2010), along with how music reward relates to other types of reward processing (Kringelbach, 2005; Berns et al., 2010; Mas-Herrero et al., 2014). The progress made with this work to quantify the heterogeneity and definition of musical “preference” has been substantial and underscores the importance of continuing efforts at quantifying musical preference, and facilitating cross-study interpretation. The music neuroscience literature would potentially be enhanced by further development of ways to quantify preference, particularly as they relate to the biological underpinnings of reward processing.

In the current study, we set out to determine if auditory stimuli that are dynamic and highly individualized, as with music (e.g., Blood and Zatorre, 2001), could be characterized by RPT, both for individual preferences and any lawful mathematical patterns. To address these questions, we adapted the RPT keypress paradigm to quantify relative preferences toward excerpts of seventeenth to twenty-first century western art music (Classical) and twentieth to twenty-first century jazz and Popular music (Popular). Our hypotheses were as follows: (1) Subjects' patterns of keypress responses would show the three types of lawful patterns observed previously with visual stimuli (Breiter and Kim, 2008; Kim et al., 2010; Lee et al., 2015). (2) These three types of patterns would be “discrete” and “recurrent” for a broad range of music categories, and exhibit scaling between individual subjects and group data. (3) Broad individual differences in the relative ordering of data points on these graphs would be observed for music preference, despite any lawful structure of the graphs fitting the data.

## Materials and methods

### Participants

This study consisted of two phases, a pilot phase and a primary experiment phase, each phase with a separate cohort. Sixteen adults between the ages of 18 and 36 with a mean (±SD) age of 27.93 ± 5.54 years participated in the pilot experiment (6 male and 10 female). Sixty-two adults participated in the primary experiment (25 males and 37 females), with an age range between 19 and 40, and a mean (±SD) age of 27.81 ± 5.99 years. Subjects from both cohorts were recruited by advertisements from the greater Chicago region through online and community placed advertisements and research registries maintained by the Department of Psychiatry and Behavioral Sciences at Northwestern University Feinberg School of Medicine and the Northwestern University Clinical and Translational Sciences Institute. Subject recruitment stopped after a set temporal window for recruitment, where target recruitment sought 40–50 subjects who completed both the Classical and Popular music keypress task, and whose data were complete. This resulted in 62 subjects participating in the study. Of the 62 participants, 49 completed both the Classical and Popular music keypress tasks and produced complete data files, meeting criteria to be included as subjects for analysis of the primary experiment. Of the 62 participants, 57 completed a questionnaire on musical experience. Fifteen reported no musical training. Of the 42 who reported some form of musical training, 14 rated themselves as beginners, 13 as intermediates, 10 as amateurs and 5 as professionals. Recruited subjects were compensated $15 per hour and provided with round trip transportation on public transport when applicable. Subjects completed this 1-h study as part of a larger suite of experiments (including experiments not presented as part of this music study) conducted over 2 days, for ~2 h each day. For additional information on participant screening procedures and demographics, please see the Supplemental Material available online.

This study was approved by the Institutional Review Board of Northwestern University and was conducted in accordance with the Declaration of Helsinki. As part of the process of consent, participants were given a printed copy of the consent form to read at their leisure. In addition, we went through each section of the consent form, and each phase of the experiment was explained to them, including all potential risks and benefits. We also reviewed the section of the consent form that included appropriate contacts for questions or concerns at that time or in the future. At the end of this meeting, participants were given the options to sign the consent form at that time, to sign it at a later date, or to choose to not participate.

### Music keypress task

The music keypress task quantified the amount of effort in terms of keypresses that subjects were willing to trade for listening time to Classical and Popular music of different categories, allowing quantification of valuation. Valuation reflected the valence of change in stimulus exposure (positive = increased exposure, negative = decreased exposure, or neutral = no change). It further reflected the magnitude of effort exerted to change the amount of exposure, quantified through the number of keypresses. Subjects thus had a choice between four possible behaviors: they could (a) approach the stimulus (keypress toward longer listening time), (b) avoid the stimulus (keypress toward shorter listening time), (c) neither approach nor avoid the stimulus (i.e., do nothing and accept the default listening time), or (d) both approach and avoid the stimulus (for example, if they overshot or undershot their desired listening time or changed their mind partway through the stimulus). This music task used procedures and analyses performed and reported previously with visual stimuli, including pictures of average and beautiful faces (Aharon et al., 2001; Elman et al., 2005; Makris et al., 2008), pictures of emotional facial expressions (Strauss et al., 2005; Perlis et al., 2008; Gasic et al., 2009), food stimuli and the International Affective Picture System or IAPS (Kim et al., 2010). The aim of the procedure was to estimate, on a subject-wise and group-wise basis, the patterns of approach or avoidance to the music stimuli.

**Figure 2:**
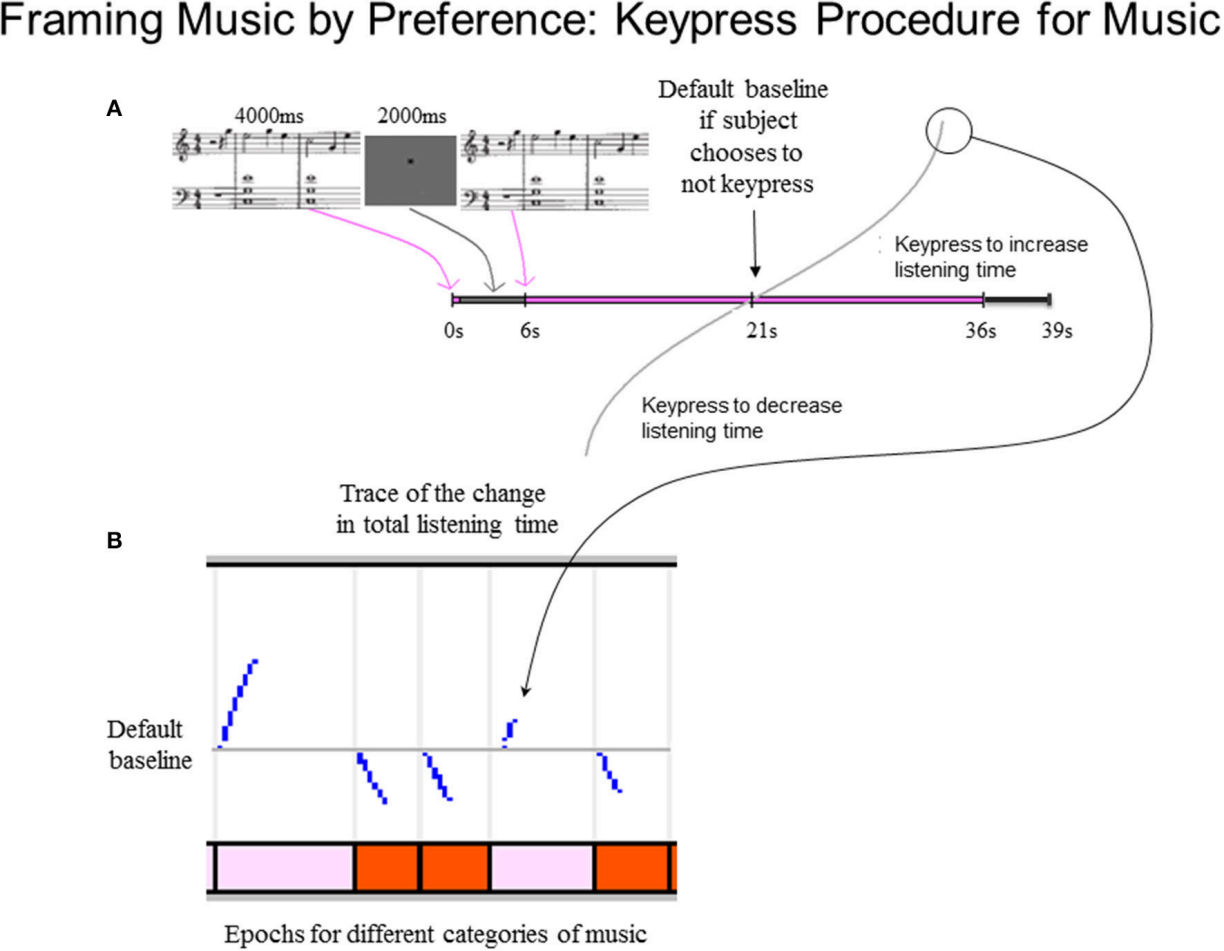
**Schematic of music preference keypress task. (A)** Keypress trial design. Each trial began with a 4-s passive pre-listening stage to allow initial assessment of the musical excerpt, followed by a 2-s silent period. The silent period was immediately followed by the active keypress listening stage during which the musical excerpt was presented. This stage had a default listening time of 15 s, but ranged in duration from 9 to 30 s on each trial depending on the keypress behavior of the subject to approach or avoid the stimulus. A 3-s masking phase was presented immediately following the active listening stage; during this stage, white noise was presented to minimize carry-over effects between trials. Subsequent trials began immediately following the end of the masking phase of the preceding trial. **(B)** Example traces of keypressing effects on listening duration during an example sequence of five trials. The vertical axis of the top panel depicts the divergence of the total trial-wise listening durations from the default baseline (15 s + 6-s pre-listening stage and 3-s post-listening masking phase). Jumps in the listening time occur in response to discrete keypress responses and adhere to a resistive function (Methods). The horizontal axis denotes the elapsed time in each trial. Colored bars in bottom panel denote the times of presentation of individual musical excerpts comprising individual trials on the keypress task.

The keypress paradigm for each of the two stimulus sets (i.e., Classical and Popular music) took about 30 min to complete. Each trial included stimulus load time (2 s, and part of the intertrial interval), a passive pre-listening stage (4 s) to allow initial assessment of the piece, and a delay to further facilitate assessment of the piece (2 s; Figure 2). These were followed by the active keypress listening stage, which had a default listening time of 15 s for each stimulus if no keypresses were made. Hence, as schematized in Figure 2, the default listening time for a trial after the 4-s passive pre-listening stage and the 2-s silent period was a total of 21 s. Subjects could decrease listening time via active avoidance keypressing close to the onset of the active keypress listening stage, or out to 30 s (30 s + 6 s = 36 s mark in Figure 2). Each trial was followed by a white noise mask (3 s), which minimized carry-over effects between trials. The total experiment duration per stimulus set had a minimum (i.e., maximum decrease keypressing) of 16 min, and a maximum (i.e., maximum increase keypressing) of 32 min and 48 s.

The pilot experiment used only classical music stimuli, and in parallel with the prior visual paradigms, included six categories of classical music with eight musical stimuli per category (see Stimuli section for additional information). Following acceptable results from the pilot phase (i.e., that music stimuli evoked relative preference graphs similar to those observed with visual stimuli), the experimental paradigm was refined in the primary experiment such that the stimulus presentation was quasi-randomized, and an additional keypress task with Popular music stimuli was added. For the primary experiment, the Classical and Popular stimulus sets were presented on separate days, and the order of presentation across the 2 days (i.e., Classical first or Popular first) was counterbalanced across subjects. For further details on experimental procedures, please see the Supplementary Material available online.

Each keypress task was implemented in Matlab and run on a personal computer in a quiet room designated for experimental testing. The hardware setup included a Lenovo X300 Think Pad with SoundMax HD Audio running Windows XP, with Sony MDRZX110 ZX headphones and a ViewSonic 22' LED monitor set to 1,440 × 900 resolution with 16 bit color, and a full-sized keyboard. The keyboard was modified by fixing a white sticker sized to cover the “C,” “V,” and “B” keys, so that subjects could find the “Z” and “X” and “N” and “M” keys with ease. Prior to the keypress task, subjects performed a keypress speed test to familiarize themselves with the alternating keypress skills. They were asked to use their writing hand to press the “Z” and “X” key in an alternating fashion as quickly as possible. Participants were given visual feedback on their keypress speed through a dynamic bar in the middle of the screen, and were asked to increase their keypress speed if they observed it dropping. They initially performed a short (10 s) practice keypress speed test to familiarize themselves with the task, and then a longer 30 s keypress speed task.

The participant's writing hand was employed using an alternating two-key keypress design (in contrast to a single keypress design) to avoid fatigue and to enable subjects to attain a high keypress speed throughout the duration of the task. The keypress task was designed to capture intrinsic motivation (Deci and Ryan, 1985; Bandura, 1997). The objective of adopting this framework was so that the more strongly participants' responses reflected their true relative preferences, the less their responses reflected random noise on the one extreme, or completely stereotyped responses (i.e., identical responses across all stimuli) on the other extreme. The ability to distinguish valid keypress-based preference behavior from subjects actively attending to the task from keypress behavior arising from a lack of task engagement was an important consideration in our experimental design. In previous work we rigorously considered the extent to which we could discriminate between such behaviors (Breiter and Kim, 2008; Kim et al., 2010). Namely, we investigated whether subjects' keypress response patterns reflect their true preference behavior (e.g., as distinguishable from random noise), and found that value functions fit from subjects' actual response data lie intermediate between value functions obtained from random noise or stereotyped (uniform) responses. We interpret these results as indirect indicators of subject task engagement and vigilance on the keypress-based preference task (Breiter and Kim, 2008; Kim et al., 2010).

In our keypress paradigm, participants have the option to do “nothing” and accept the default listening time, or alternatively to modulate how long the stimulus is presented by actively keypressing. The patterns of their responses are quantified by RPT, but individuals not engaging in the task who either never keypress or respond identically to every music stimulus will exhibit unquantifiable keypress entropy (see *Relative Preference Analysis*, Methods), and thus will produce a data for which no valid value function can be computed. Likewise, subjects who theoretically make completely random keypress responses regardless of the presented stimulus would tend to generate data lying far removed from the distribution of value functions observed in subjects performing the task (see preceding paragraph). In such cases, observing the subject's value function (if computable at all) conforming to the distribution of value functions from the rest of the study sample is useful as an indirect fidelity measure for such an individual's low level of engagement. However, we emphasize that there is no way to absolutely rule out the possibility that subjects were not attending to the music stimuli and responding in ways that do not reflect their true musical preference. To minimize this possibility, we informed all participants that the duration of the experiment was independent of their keypress behavior. Subjects were told this to reduce the possibility that their keypress behavior would be influenced by a desire to finish the experiment more quickly. Therefore, from the subjects' perspective, their ability to exert control involved prolonging exposure to stimuli they enjoyed the most, and minimizing exposure to stimuli they enjoyed least. In order to remain objective, we had to accept subjects' keypress behavior at face value rather than making subjective assumptions about what sorts of keypress behavior would or would not reflect sufficient vigilance to the task. Anecdotally, our experience across multiple studies (Aharon et al., 2001; Strauss et al., 2005; Makris et al., 2008; Perlis et al., 2008; Gasic et al., 2009; Kim et al., 2010; Lee et al., 2015; Viswanathan et al., 2015, 2017) has been that subjects not attending to keypress tasks tend to make stereotyped keypress responses with similar avoidance values to all presented stimuli (i.e., to reduce overall viewing time) (Kim et al., 2010). Such subjects are excluded from analysis due to the inability to compute keypress entropy and thereby fit a value function. This was very rarely encountered in our prior experiments, nor was it seen in the current study, as indicated by the rare incidence of subject exclusion (see *Preliminary Analysis* and *Relative Preference Analysis*, Methods, and Supplementary Material).

For further details on experimental procedures and tables of valid keypress responses across all datasets, please see Supplementary Material available online.

### Musical stimuli

The seventeenth to twenty-first century western art music (Classical) stimulus set consisted of six categories in the Western art music tradition, based on agreed upon time periods in music scholarship (Baroque [years 1600–1750], Classical [years 1751–1814], Romantic [years 1815–1895], Early twentieth Century [years 1896–1945], Late twentieth Century [years 1946–1999], twenty-first Century [years 2000–present]). These stimuli comprised eight excerpts (4 vocal, 4 instrumental) in each category for a total of 48 excerpts. The 48 music excerpts were selected by a professional music theorist (AEB). For further details on criteria for choosing these music excerpts, please see the Supplementary Material available online.

The 48 Classical music excerpts were chosen based on these criteria: (i) the excerpt was composed within the time period, (ii) the composer is often considered to represent that time period and its stylistic features (e.g., Bach represents and is a currently well-known composer of the Baroque time period while Mozart represents and is a well-known composer of the Classical time period), (iii) the excerpt represents stylistic features of the time period as agreed upon in music scholarship (e.g., representative Classical music often uses diatonic harmonies whereas representative Romantic music often uses chromatic harmonies). In addition, to ensure each piece had sufficient volume during the experiment, each excerpt was linearly transformed, so that each piece had the same maximum loudness. Of the 48 Classical excerpts, 24 contained vocals and 24 were instrumental.

The twentieth to twenty-first century jazz and Popular music (Popular) stimulus set also consisted of six categories (Alternative, Metal, Adult, Classic Rock, Jazz Fusion, and Classic Jazz), again with eight excerpts in each for a total of 48 excerpts. The categories of Popular music were chosen to optimize between-category variation. Within each category, we aimed to pick excerpts that were famous and others that were less well-known but reflected similar musical characteristics. For the Popular music stimulus set, there were never more than two excerpts from the same band within a genre. In addition, we chose some more specialized categories such as heavy metal for their polarizing nature, as well as two broad eras of jazz music. Classic jazz includes artists from the bebop (e.g., Charlie Parker and John Coltrane) and cool jazz eras (e.g., Miles Davis, Dave Brubeck). We also include jazz fusion titles from the 1970s (e.g., Chick Corea, Pat Metheny, Jean-Luc Ponty). Of the 48 Popular music excerpts, 11 contained vocals and 37 were instrumental.

For the pilot experiment, the 48 Classical excerpts were presented in a fixed order. For the primary experiment, the paradigm was refined and the 48 trials in each task (e.g., Classical and Popular music) were quasi-randomized. Quasi-randomization was obtained by using a Matlab random seed generator that produced a random ordered non-replenishing integer between 1 and 48, which mapped to each of the 48 excerpts per stimulus set. Task order (i.e., Classical vs. Popular music) was counterbalanced across subjects.

### Preliminary analysis

Preliminary analysis of data integrity was performed on keypress data from all 78 subjects. Of the 78 subjects, 8 subjects were excluded from the analysis because they failed to return to complete the second half of the experiment. Another 5 subjects were excluded due to corrupt (*N* = 1) or partial (*N* = 4) data sets. A total of 114 datasets were used for analysis from 16 subjects for the pilot and 49 subjects for the primary study.

The remaining valid data sets (16 for the pilot; 49 for Classical and Popular music) were evaluated using range (min, max), location (mean) and dispersion measures (standard deviation) for the numbers of keypresses increasing viewing time (*K*^+^) and the numbers of keypresses decreasing viewing time (*K*^−^), and were deemed normally distributed valid data.

In addition, as an exclusion criterion, the raw data were evaluated for cases when *K* = 0 for a given category (i.e., cases where the subject made no keypresses to either approach or avoid any stimulus in the category). Computing the Shannon entropy (*H*) for a given music category requires that *K* > 0; therefore, *H* is undefined for categories in which the subject does not keypress for any of the stimuli. In such a situation (i.e., *K* = 0), the *H* computation results in evaluating log(0/0), which is undefined, necessitating data exclusion.

Furthermore, to fit models to our data, the data had to be screened for additional inclusion criteria. The model fit inclusion criteria were as follows: (1) valid entropy (*H*) calculations (see prior paragraph); (2) sufficient number of data points to fit the model with a computable *R*^2^; and (3) coherence of model fits between individual and group data. This last criterion means that the curve direction of individual subject model fits must be consistent with the curve direction of the group level boundary envelopes, and therefore corroborate the majority of the observed subject data. Altogether, there were 6 types of model fitting, which included group level boundary envelopes (power-law fits with offsets and logarithmic fits for group *(K, H)* value functions, and quadratic fits for group *(K*, σ*)* limit functions) in addition to logarithmic and simple power-law fits for individual (*K, H)* value functions, and quadratic fits for individual *(K*, σ*)* limit functions. Model fit analysis of individual subject data resulted in 326 potential fits for each model, including 32 data sets for the pilot, and 98 for Classical, 98 for Popular music and 98 for pooled Classical and Popular music data. Model fit analysis for group data produced 2 fits for each of 3 models fit to 4 different datasets, leading to 24 total model fits for the group level analyses. Overall, our reported data contained a total of 1,002 potential model fits. Of the 1,002 potential model fits, a total of 103 data sets were excluded. For group level fitting of boundary envelopes, there were 0/24 fits excluded. For logarithmic fit modeling of individual (*K, H*) data, there were 15/326 data sets excluded. For the simple power-law fit modeling of individual (*K, H*) data, there were 58/326 data sets excluded. Finally, for the quadratic fit modeling of individual *(K*, σ*)* data, there were 30/326 data sets excluded. Model exclusions for individual subjects are tabulated in Tables S1–S5, and detailed for each analysis in the Supplementary Results Material available online.

### Relative preference analysis

In carrying out the relative preference analysis, we followed the methodology described in detail in Kim et al. (2010). We specifically used an iterative modeling approach (Banks and Tran, 2009) in which we sought to identify RPT patterns in the data and three signatures of potential lawfulness, as done previously with visual stimuli (Breiter and Kim, 2008; Kim et al., 2010). This meant observing “discrete” mathematical fitting of patterns within the data, “recurrence” of patterns across different stimulus sets and experiments, and “scalability” of the observed patterns. We utilized the datasets that met stringent criteria for quality assurance, and assessed the graphical structure between the following variables: mean numbers of keypresses to approach or avoid stimuli within a music category (*K*^+^, *K*^−^), the Shannon entropy of approach/avoidance keypressing within a category (*H*^+^, *H*^−^), and the standard deviation (σ^+^, σ^−^) of approach or avoidance keypressing within a category. Graphical analysis sought to determine the presence of functions, manifolds, or boundary envelopes to individual, and separately, group data that followed the same form as RPT functions, manifolds, and boundary envelopes (Breiter and Kim, 2008; Kim et al., 2010; Lee et al., 2015).

#### (*K, H*) value functions

The first relationship we considered was the relationship between mean keypresses to approach or avoid across stimuli within a music category (*K*^+^, *K*^−^) and the Shannon entropy of approach/avoidance keypressing (*H*^+^, *H*^−^). We used the following approach to compute the Shannon entropy separately for the positive (approach) and negative (avoidance) keypress responses in each category. First, consider an ensemble of the numbers of either approach or avoidance keypress responses (i.e., numbers of keypresses) *A* across stimuli within a single music category: *A*^±^ = *(a*_1_*, a*_2_*, …, a*_*n*_*)*. We can then define the relative proportions of the approach or avoidance keypress responses for the individual stimuli, *p*_*i*_, such that:

(1) $$\begin{array}{r} p_{i}=a_{i}/\overset{N}{\underset{j=1}{\sum }}a_{j}. \end{array}$$

Using these proportions of the keypress responses, the Shannon entropy of the keypress response pattern can be computed for an individual music category as follows:

(2) $$\begin{array}{r} H^{\pm }=\underset{i}{\sum }p_{i}\log_{2}\frac{1}{p_{i}}. \end{array}$$

After computing the values of *K*^±^ and *H*^±^ for each music category, (*K, H*) value functions can be generated by plotting the Shannon entropy *H*^±^ against the mean keypresses *K*^±^ for all music categories in an individual subject. (*K, H*) data were also plotted across multiple subjects to visualize data at the group level.

At the group level, we assessed if (*K, H*) data contained boundary envelopes that conformed well to either logarithmic functions (*H* = *a* log_10_ *K* + *b*) or power-law functions with offsets (*H* = (*K* + *a*)^*b*^ + *c*); see the Supplementary Material available online for details. At the individual subject level, we assessed fits for logarithmic (*H* = *a* log_10_ *K* + *b*) and simple power-law (*H* = *b K*^*a*^) functions to the (*K, H*) data for approach and avoidance across music categories for individual subjects (e.g., **Figures 4A,C**). The fits for logarithmic and power-law functions were achieved by performing simple linear regression on *H* vs. log_10_ *K*, and log_10_ *H* vs. log_10_*K*, respectively.

#### (*K*, σ) limit functions

The second relationship considered was that between the mean keypresses toward approach or avoidance (*K*^±^) and the standard deviation of approach or avoidance keypressing across stimuli within a music category (σ^±^). (*K*, σ) limit functions were generated by plotting values of σ against *K* for all music categories either in an individual subject or pooling the data together across subjects in a group analysis. At both the individual and group level, we found that (*K*, σ) limit functions were well characterized by quadratic functions of the form σ = *a K*^2^ + *b K* + *c*. For the group data, we fit quadratic boundary envelopes to the (*K*, σ) data much in the same manner performed for the (*K, H*) value functions (see Supplementary Material available online for further details). For individual subject analysis, we fit quadratic functions directly to the (*K*, σ) data using the *polyfit* function in Matlab.

#### (*H*^+^, *H*^−^) trade-off plots

(*H*^+^, *H*^−^) trade-off (or opponency) plots were defined by plotting the Shannon entropy for approach (*H*^+^) against the Shannon entropy for avoidance (*H*^−^) for all music categories in a given stimulus set. These plots were generated either across music categories for an individual subject, or by pooling data across all subjects in the cohort to generate a group-level plot. For both the individual subject and group-level data, we found that (*H*^+^, *H*^−^) data conformed to a radial distribution about the origin of the trade-off plot, such that $r=\sqrt{{\left(H^{-}\right)}^{2}+{\left(H^{+}\right)}^{2}}$, or, equivalently, $H^{+}=\sqrt{r^{2}-{\left(H^{-}\right)}^{2}}$. Radial fits were estimated for individual subjects as well as the group-level data by computing the mean radial distance, *r*, across all (*H*^+^, *H*^−^) data in the trade-off plot.

#### Relative ordering of categorical preferences

Using methods developed to assess relative preference logic in Kim et al. (2010), the relative rank orderings of Classical and Popular music categories along the (*K, H*) value function were measured for each individual, considering both logarithmic and power-law fits. To determine rank order, the (*K, H*) values for individual music categories were projected onto the fitted value function by identifying the point on the value function with the shortest distance to the observed (*K, H*) data. Then, the relative order of music categories on the (*K, H*) curve was computed for each individual, beginning with music categories falling nearest to the origin of the value function. Determining the relative ordering of music categories for each individual subject allowed us to assess whether, across stimulus sets and experiments, there were multiple subjects with the exact same rank ordering of music categories by preference. This data was tabulated in frequency histograms to show the numbers of subjects who share rank orderings in common.

## Results

### Classical music pilot experiment

#### Group-level (*K, H*), (*K, σ*), and (*H*^+^, *H*^−^) analyses

**Figure 3:**
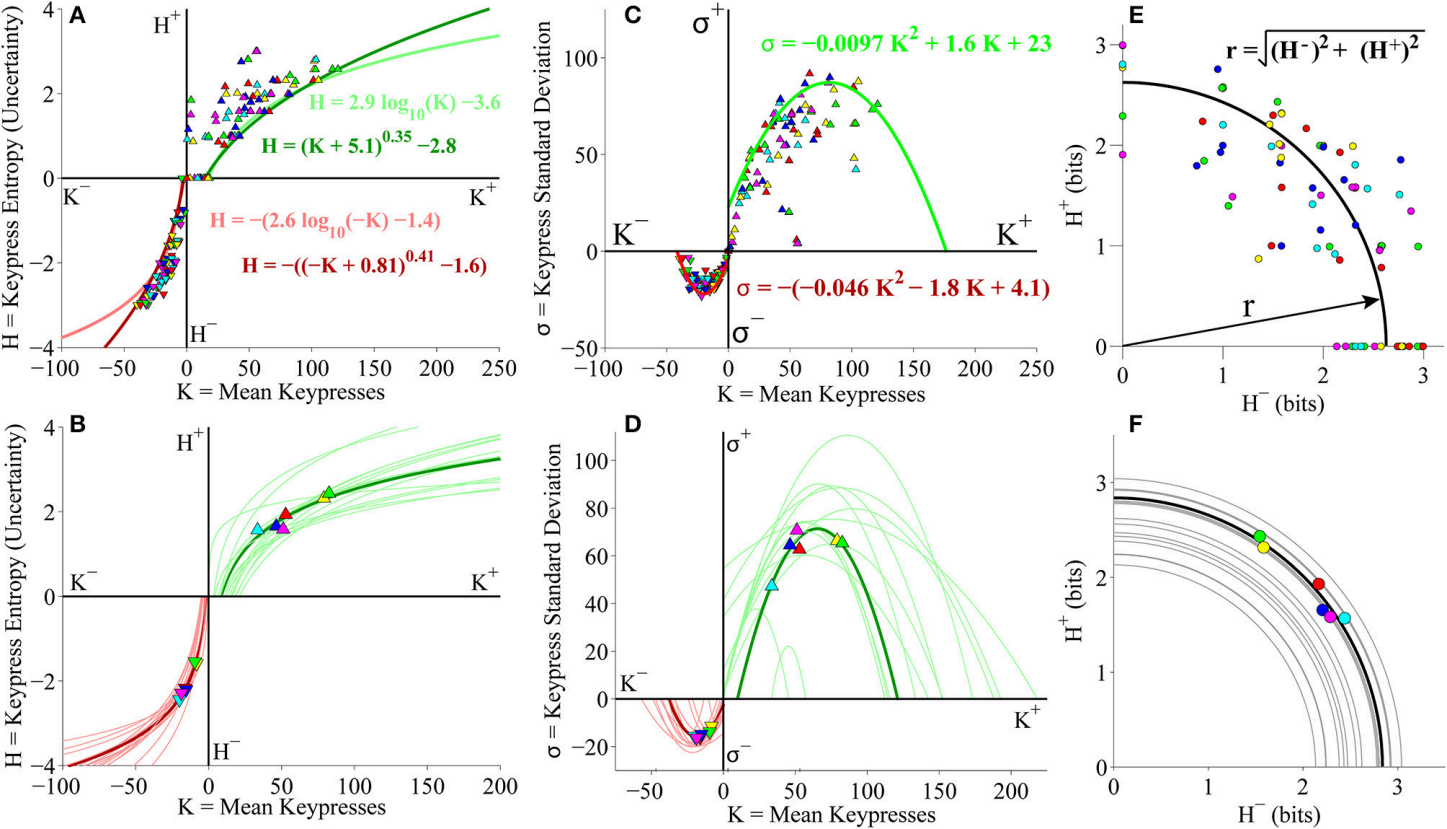
**Relative preference results for pilot Classical music experiment. (A)** Group value functions for (*K, H*) data. Symbols denote individual (*K, H*) data pairs for approach and avoidance keypressing across musical categories for all subjects in cohort. Boundary envelopes to the approach and avoidance keypressing data were well fit by logarithmic functions (light green/red) or power-law functions with an offset (dark green/red). **(B)** Value functions comparing mean keypress intensity (*K*) to keypress entropy (*H*) in individual subjects. Symbols indicate *K* and *H* values computed within six Classical music categories for either approach (*K*^+^, *H*^+^) or avoidance (*K*^−^, *H*^−^) keypressing behavior within a single representative subject. Each color denotes a Classical music category. Upward-pointing symbols correspond to approach keypressing; downward-pointing symbols correspond to avoidance keypressing. Dark green and red traces indicate logarithmic fits to approach and avoidance data for the representative subject; superimposed light green and red traces indicate individual log fits for remaining subjects in the cohort. **(C)** Group limit functions for (*K*, σ) data. Boundary envelopes to (*K*, σ) were well fit by quadratic functions (red and green traces). **(D)** Limit functions comparing *K* to the standard deviation of approach or avoidance keypressing (σ) across Classical music categories in individual subjects. Approach and avoidance data for individual subjects were fit to quadratic functions, such that σ = *a K*^2^ + *b K* + *c*. **(E)** Group trade-off plot for (*H*^+^, *H*^−^) data. Black line indicates radial fit computed for (*H*^+^, *H*^−^) pairs across all musical categories and all subjects in the cohort. **(F)** Trade-off plot comparing entropy for approach (*H*^+^) and avoidance (*H*^−^) keypressing across Classical music categories in individual subjects. Symbols denote (*H*^+^, *H*^−^) data pairs for each music category in the representative subject. Black line denotes radial fit of (*H*^+^, *H*^−^) pairs computed for representative subject, such that *r* = $\;\sqrt{{\left(H^{+}\right)}^{2}+{\left(H^{-}\right)}^{2}}$; gray lines denote radial fits for remaining subjects in cohort.

We evaluated the distributions between keypress mean, standard deviation and pattern variables at the group level, by pooling data across all music categories and all subjects. As a preliminary analysis, we considered approach and avoidance keypressing behavior within an initial cohort of 16 subjects keypressing to six categories of Classical music. We first examined the relationship between mean keypresses to approach or avoid across stimuli within a music category (*K*), and the Shannon entropy of the approach/avoidance keypresses within a category (*H*; Methods; Figures 3A,B). Next, we examined the relationship between *K* and the standard deviation of keypress responses within categories (σ; Figures 3C,D). Finally, we considered the relationship between avoidance entropy for each category (*H*^−^) and the approach entropy for each category (*H*^+^), which we refer to as the trade-off plot (Figures 3E,F).

Figure 3A displays group data comparing mean keypresses (*K*) for each subject and music category to the keypress entropy (*H*). We fit boundary envelopes to the group (*K, H*) data by estimating the outward edge of the (*K, H*) distribution and then fitting either a logarithmic function (*H* = *a* log *K* + *b*) or a power-law with vertical and horizontal offsets (*H* = *(K* + *a)*^*b*^ + *c*; Methods). Both logarithmic (light traces) and power-law (dark traces) functions effectively approximated the shape of the boundary envelope (Figure 3A), as with the individual subject fits (Figure 3B). We similarly fit boundary envelopes to the group limit function comparing *K* and σ across music categories (Figure 3C). The boundary envelopes for (*K*, σ) data were fit using quadratic functions, as for the individual subject fits (Figure 3D). In addition, the group (*H*^+^, *H*^−^) data exhibited a radial distribution around the origin of the trade-off plot (mean radial distance = 2.63 bits), albeit with greater dispersion of the radial distance across subjects when analyzing data at the group level (Figure 3E) than observed when considering fits within individual subjects (Figure 3F).

#### Individual subject (*K, H*) value functions

Individual subjects' (*K, H*) value functions were well fit by logarithmic functions of the form *H* = *a* log *K* + *b* (Figure 3B). Figure 3B illustrates these fits for a representative subject (dark traces; symbols indicate (*K, H*) values for individual music categories) and for the remaining subjects in the cohort (light traces). All subjects demonstrated the same logarithmic shape for both approach and avoidance curves. Subjects' value functions were also well fit by simple power-law functions of the form *H* = *b K*^*a*^ (not shown). Table 1 provides goodness of fit estimates for the logarithmic (log *K, H*) and power-law (log *K*, log *H*) fits to (*K, H*) value functions.

**Table 1:** **Mean goodness of fit statistics for value and limit functions for music categories for the pilot experiment Classical music stimulus set**.

**Variables**	**Parameter**	**Mean ± SD for parameters**
(Log *K*^−^, *H*^−^)	*R*^2^	0.91 ± 0.11
	Adjusted *R*^2^	0.89 ± 0.14
	*F* statistic of regression	396 ± 613
	*p*-value of regression	0.017 ± 0.031
(Log *K*^+^, *H*^+^)	*R*^2^	0.85 ± 0.17
	Adjusted *R*^2^	0.80 ± 0.22
	*F* statistic of regression	421 ± 903
	*p*-value of regression	0.040 ± 0.071
(Log *K*^−^, Log *H*^−^)	*R*^2^	0.85 ± 0.22
	Adjusted *R*^2^	0.80 ± 0.33
	*F* statistic of regression	104 ± 97
	*p*-value of regression	0.050 ± 0.16
(Log *K*^+^, Log *H*^+^)	*R*^2^	0.76 ± 0.29
	Adjusted *R*^2^	0.66 ± 0.39
	*F* statistic of regression	75 ± 107
	*p*-value of regression	0.13 ± 0.18
(*K*^−^, σ^−^)	*R*^2^	0.85 ± 0.18
	Adjusted *R*^2^	0.74 ± 0.30
	*F* statistic of regression	160 ± 534
	*p*-value of regression	0.085 ± 0.15
(*K*^+^, σ^+^)	*R*^2^	0.82 ± 0.27
	Adjusted *R*^2^	0.70 ± 0.45
	*F* statistic of regression	89 ± 149
	*p*-value of regression	0.12 ± 0.24

#### Individual subject (*K*, σ) and (*H*^+^, *H*^−^) plots

Consistently, subjects exhibited a quadratic shape to their (*K*, σ) curves, of the form σ = *a K*^2^ + *b K* + *c*. σ began small for low values of *K*, at which point σ quickly rose to a peak value at intermediate *K* values, before finally declining and returning to baseline levels (Figure 3D). The same trend was observed for the approach and avoidance curves in this regard, although the avoidance curves achieved much smaller peak heights at lower magnitudes of *K*. Table 1 provides goodness of fit estimates for the quadratic fits to (*K*, σ) limit functions.

Plotting the relationship between *H*^−^ and *H*^+^ is informative because it reveals tendencies of the subject toward approach vs. avoidance (depending on the polar angle) as well as indifference vs. conflict (depending on the radial distance) for each category (Kim et al., 2010). Subjects showed clear tendencies toward a radial distribution of (*H*^+^, *H*^−^) data in the trade-off plot, such that the radial distances of the (*H*^+^, *H*^−^) pairs were positioned roughly a constant distance from the origin across music categories. Figure 3F displays the distribution of (*H*^+^, *H*^−^) pairs and the mean radial fit for the representative subject (black trace), in addition to those for the remaining subjects in the cohort (gray traces).

### Primary classical music experiment

#### Group-level (*K, H*), (*K*, σ), and (*H*^+^, *H*^−^) analyses

**Figure 4:**
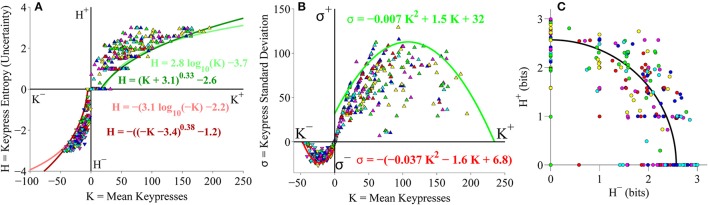
**Group relative preference results for the Classical music stimulus set in the primary experiment. (A)** Approach and avoidance boundary envelopes for (*K, H*) data were well fit by logarithmic functions (light red/green traces) and power-law functions with offset (dark red/green traces). Symbols depict (*K, H*) data pairs for the six individual Classical music categories across all subjects in the cohort. **(B)** Boundary envelopes for the group (*K*, σ) data were well fit by quadratic functions (green/red traces). Symbols depict (*K*, σ) data pairs for all Classical music categories and subjects. **(C)** Trade-off plot showing group (*H*^+^, *H*^−^) data. Symbols indicate (*H*^+^, *H*^−^) data pairs for each individual music category within individual subjects. Black line indicates radial fit computed across all subjects and music categories, such that $r\;=\;\sqrt{{\left(H^{+}\right)}^{2}+{\left(H^{-}\right)}^{2}}$.

After establishing these consistent patterns between mean keypress behavior and pattern variables of keypressing for the pilot Classical music stimulus set (Figures 3A,C,E), we repeated these analyses for the primary experiment involving a larger cohort (49 subjects) with data available for categories of both Classical and Popular music, and a randomized order of stimulus presentation. We first considered the Classical and Popular music stimulus sets separately. Looking at group keypressing data across six Classical music categories in this larger cohort, we replicated the same findings as observed in our pilot phase cohort. Boundary envelopes for group (*K, H*) value functions were again well fit by logarithmic and power-law (with vertical and horizontal offsets) functions (Figure 4A), while boundary envelopes for group (*K*, σ) limit functions were well fit by quadratic functions (Figure 4B). Group-level (*H*^+^*, H*^−^) data exhibited a radial distribution about the origin of the trade-off plot, as observed previously, with a mean radial distance of 2.57 bits (Figure 4C).

#### Individual subject (*K, H*) value functions

**Figure 5:**
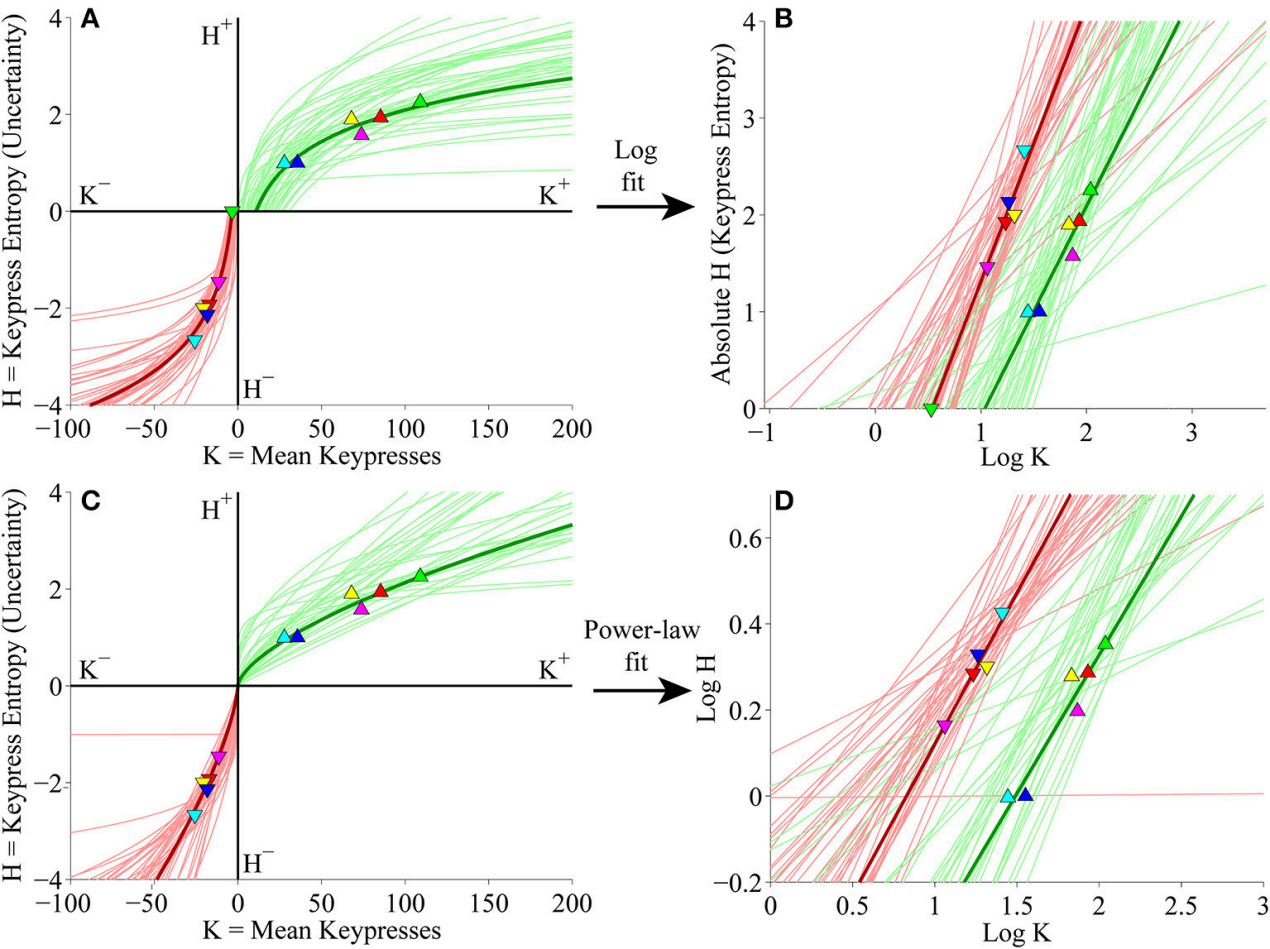
**Individual (*K*, *H*) value functions and (*K*, *H*) regressions for the Classical music stimulus set in the primary experiment. (A)** Logarithmic fits to (*K, H*) data within individual subjects. Linear regression of *H* vs. log_10_ *K* for the approach and avoidance data (separately) resulted in logarithmic fits of the form *H* = *a* log_10_ *K* + *b*. Symbols indicate (*K, H*) values within individual music categories for approach and avoidance keypressing in a representative subject. Dark green/red traces indicate log fits for approach/avoidance keypressing in the representative subject; light green/red traces indicate log fits for remaining subjects of the cohort. **(B)** Semilog plot illustrating linear regression of *H* vs. log_10_ *K* for approach (green) and avoidance (red) data in the representative subject (dark traces) and remaining subjects in cohort (light traces). This linear regression procedure produced the log fits depicted in **(A)**. **(C)** Simple power-law fits to (*K, H*) data within individual subjects. Linear regression of log_10_ *H* vs. log_10_ *K* resulted in power-law fits of the form *H* = *b K*^*a*^. **(D)** Log-log plot illustrating linear regression of log_10_ *H* vs. log_10_ *K* for the approach and avoidance data in individual subjects. This linear regression produced the power-law fits depicted in **(C)**.

We next considered keypress variables in the primary experiment for Classical music categories at the individual subject level. First, (*K, H*) value functions were fit separately with logarithmic functions (*H* = *a* log *K* + *b*) and simple power-law functions (*H* = *b K*^*a*^). Figure 5A depicts the logarithmic fits to individual subjects' approach and avoidance curves, highlighting the (*K, H*) data for individual music categories and fits for a representative subject (symbols, dark traces). These logarithmic fits were estimated by performing simple linear regression of *H* against log_10_ *K*. The linear fits are depicted graphically in Figure 5B, which plots *H* against log_10_ *K*, highlighting the results of regression for the representative subject (symbols, dark traces). Figure 5C displays the results of the simple power-law fits to individual subjects' (*K, H*) data. Power-law fits were estimated by performing simple linear regression of log_10_ *H* against log_10_ *K*; the results of these regressions are displayed graphically in Figure 5D. Goodness of fit estimates for logarithmic and power-law approximations to the (*K, H*) value functions for the primary Classical music analysis are provided in Table 2.

**Table 2:** **Mean goodness of fit statistics for value and limit functions for music categories for the primary experiment Classical music stimulus set**.

**Variables**	**Parameter**	**Mean ± SD for parameters**
(Log *K*^−^, *H*^−^)	*R*^2^	0.88 ± 0.14
	Adjusted *R*^2^	0.85 ± 0.17
	*F* statistic of regression	778 ± 2,232
	*p*-value of regression	0.019 ± 0.036
(Log *K*^+^, *H*^+^)	*R*^2^	0.84 ± 0.20
	Adjusted *R*^2^	0.77 ± 0.32
	*F* statistic of regression	205 ± 485
	*p*-value of regression	0.059 ± 0.13
(Log *K*^−^, Log *H*^−^)	*R*^2^	0.86 ± 0.19
	Adjusted *R*^2^	0.81 ± 0.27
	*F* statistic of regression	347 ± 1,389
	*p*-value of regression	0.041 ± 0.14
(Log *K*^+^, Log *H*^+^)	*R*^2^	0.84 ± 0.22
	Adjusted *R*^2^	0.77 ± 0.36
	*F* statistic of regression	311 ± 989
	*p*-value of regression	0.063 ± 0.15
(*K*^−^, σ^−^)	*R*^2^	0.88 ± 0.19
	Adjusted *R*^2^	0.79 ± 0.31
	*F* statistic of regression	171 ± 399
	*p*-value of regression	0.073 ± 0.15
(*K*^+^, σ^+^)	*R*^2^	0.82 ± 0.26
	Adjusted *R*^2^	0.71 ± 0.43
	*F* statistic of regression	1.8e30 ± 1.2e31
	*p*-value of regression	0.12 ± 0.23

#### Individual subject (*K*, σ) and (*H*^+^, *H*^−^) analyses

**Figure 6:**
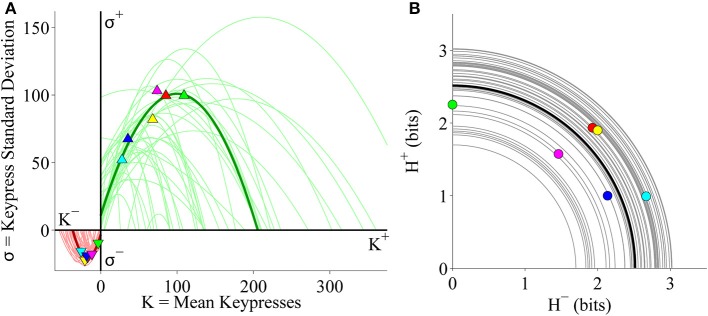
**Limit and trade-off fits for individual subjects from the Classical music stimulus set in the primary experiment. (A)** Limit functions show the relationship between mean keypressing (*K*) and keypress standard deviation (σ) across Classical music categories within individual subjects, highlighting a representative subject (symbols, dark traces). Limit functions for approach and avoidance keypressing were quadratic fits of the form σ = *a K*^2^ + *b K* + *c*. **(B)** Trade-off plot fits illustrate the relationship between avoidance (*H*^−^) and approach (*H*^+^) keypress entropy across Classical music categories, within individual subjects. Radial fits were computed for each subject such that $r\;=\;\sqrt{{\left(H^{+}\right)}^{2}+{\left(H^{-}\right)}^{2}}$. Symbols and black trace indicate data and fit for a representative subject; radial fits for remaining subjects are indicated by gray traces.

We also considered the relationships between *K* and σ as well as *H*^+^ and *H*^−^ at the individual subject level. As seen in the pilot cohort, individual (*K*, σ) limit functions were well fit by quadratic functions (Figure 6A). Table 2 indicates goodness of fit estimates for the quadratic functions fit to individual (*K*, σ) data. Also consistent with earlier results, individual (*H*^+^*, H*^−^) data were radially distributed about the origin of the trade-off plot (Figure 6B). Figure 6B demonstrates these (*H*^+^, *H*^−^) trade-off functions for individual subjects.

### Popular music experiment

#### Group-level (*K, H*), (*K*, σ), and (*H*^+^, *H*^−^) analyses

**Figure 7:**
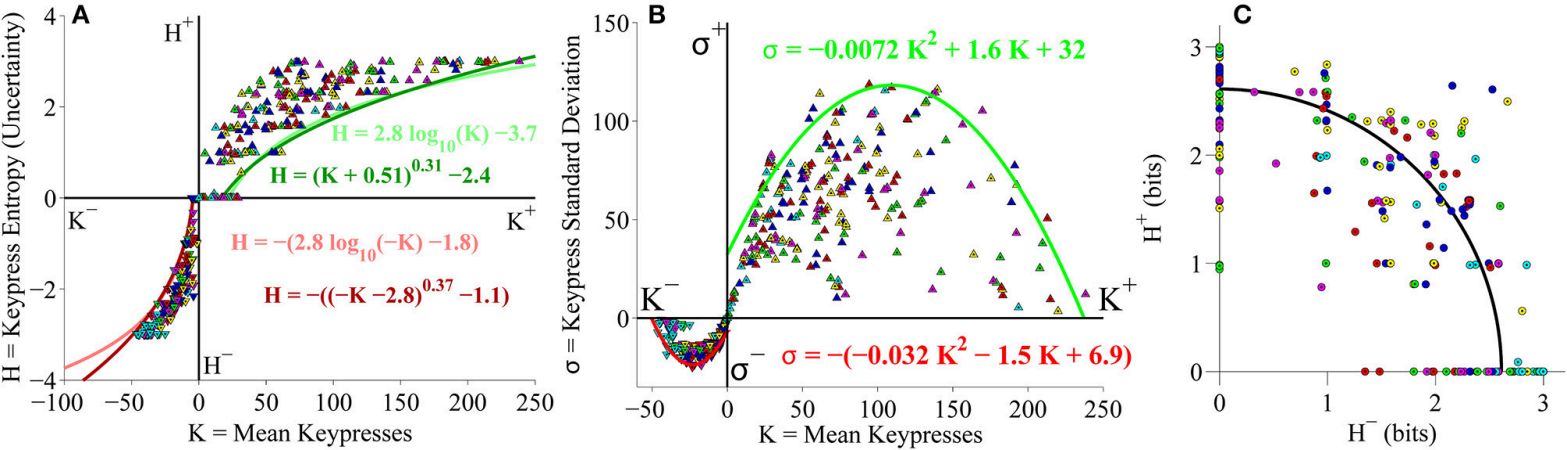
**Group relative preference results for the Popular music stimulus set in the primary experiment. (A)** Approach and avoidance boundary envelopes for (*K, H*) data were fit by logarithmic functions (light red/green traces) and power-law functions with offset (dark red/green traces). Symbols depict (*K, H*) data pairs for the six individual Popular music categories across all subjects in the cohort. **(B)** Boundary envelopes for the group (*K*, σ) data were fit with quadratic functions (green/red traces). Symbols depict (*K*, σ) data pairs for all Popular music categories and subjects. **(C)** Trade-off plot showing group (*H*^+^, *H*^−^) data. Symbols indicate (*H*^+^, *H*^−^) data pairs for each individual music category within individual subjects. Black line indicates radial fit computed across all subjects and music categories, such that $r\;=\;\sqrt{{\left(H^{+}\right)}^{2}+{\left(H^{-}\right)}^{2}}$.

After confirming that the relationships between mean keypressing and pattern variables replicated across separate cohorts of subjects keypressing to Classical music categories, we asked if these results extended to a different genre of music. We performed an identical analysis to keypressing data from the same large cohort of subjects (as depicted in Figures 4–6), but this time using keypressing data related to six categories of Popular music. We observed the same relationships between keypressing and all pattern variables evaluated in the pilot experiment. Figure 7 displays the relative preference relationships computed on the group-level data for the Popular music cohort. As seen before, group boundary envelopes could be fit to (*K, H*) value function data using either a logarithmic function or power-law with offsets (Figure 7A). Boundary envelopes fit to the group (*K*, σ) limit function data were again well fit using quadratic functions (Figure 7B), while group (*H*^+^, *H*^−^) data in the trade-off plot were radially distributed about the origin, with a mean radial distance of 2.61 bits (Figure 7C).

#### Individual subject (*K, H*) value functions

**Figure 8:**
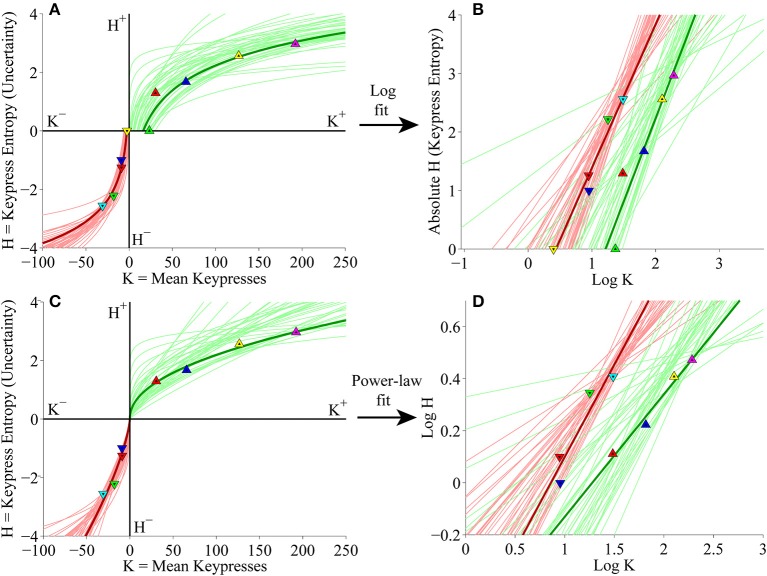
**Individual (*K*, *H*) value functions and (*K*, *H*) regressions for the Popular music stimulus set in the primary experiment. (A)** Logarithmic fits to (*K, H*) data within individual subjects. Symbols indicate (*K, H*) values within individual music categories for approach and avoidance keypressing in a representative subject. Dark green/red traces indicate log fits for approach/avoidance keypressing in a representative subject; light green/red traces indicate log fits for remaining subjects of the cohort. **(B)** Semilog plot illustrating linear regression of *H* vs. log_10_ *K* for approach (green) and avoidance (red) data in a representative subject (dark traces) and remaining subjects in cohort (light traces). **(C)** Simple power-law fits to (*K, H*) data within individual subjects. **(D)** Log-log plot illustrating linear regression of log_10_ *H* vs. log_10_ *K* for the approach and avoidance data in individual subjects.

At the individual subject level, subjects' (*K, H*) value functions were again well fit by logarithmic (Figure 8A) or simple power-law (Figure 8C) functions when keypressing to the Popular music categories. Figures 8B,D display the linear regressions of *H* against log_10_ *K* and log_10_ *H* against log_10_ *K* used to estimate the logarithmic and power-law fits, respectively.

#### Individual subject (*K*, σ) and (*H*^+^, *H*^−^) analyses

**Figure 9:**
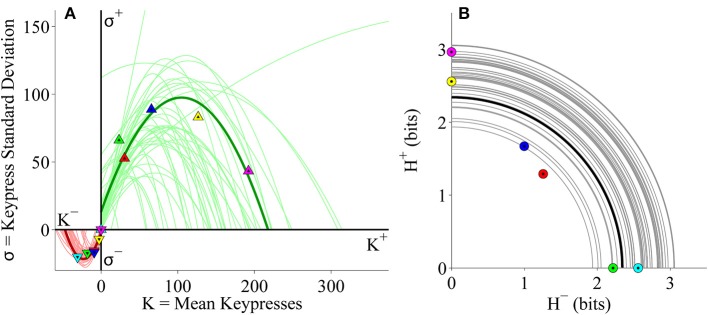
**Limit and trade-off fits for individual subjects from the Popular music stimulus set in the primary experiment. (A)** Limit functions show the relationship between mean keypressing (*K*) and keypress standard deviation (σ) across Popular music categories within individual subjects, highlighting the representative subject (symbols, dark traces). Limit functions for approach and avoidance keypressing are quadratic fits (σ = *a K*^2^ + *b K* + *c*). **(B)** Trade-off plot fits illustrate the relationship between avoidance (*H*^−^) and approach (*H*^+^) keypress entropy across Popular music categories, within individual subjects. Radial fits were computed for each subject such that *r* = $\;\sqrt{{\left(H^{+}\right)}^{2}+{\left(H^{-}\right)}^{2}}$. Symbols and black trace indicate data and fit for a representative subject; radial fits for remaining subjects are indicated by gray traces.

Individual subject results for the (*K*, σ) and (*H*^+^, *H*^−^) relationships for keypressing to Popular music categories also corroborated the results with Classical music. Individual (*K*, σ) data were well fit using quadratic functions (Figure 9A), while (*H*^+^, *H*^−^) trade-off data conformed to radial distributions about the origin (Figure 9B). Goodness of fit estimates for logarithmic and power-law fits to individual (*K, H*) data as well as quadratic fits to (*K*, σ) data for the Popular music analysis are presented in Table 3.

**Table 3:** **Mean goodness of fit statistics for value and limit functions for music categories for the primary experiment Popular music stimulus set**.

**Variables**	**Parameter**	**Mean ± SD for parameters**
(Log *K*^−^, *H*^−^)	*R*^2^	0.90 ± 0.12
	Adjusted *R*^2^	0.87 ± 0.16
	*F* statistic of regression	595 ± 1,571
	*p*-value of regression	0.021 ± 0.037
(Log *K*^+^, *H*^+^)	*R*^2^	0.88 ± 0.19
	Adjusted *R*^2^	0.84 ± 0.25
	*F* statistic of regression	1,069 ± 3,647
	*p*-value of regression	0.044 ± 0.11
(Log *K*^−^, Log *H*^−^)	*R*^2^	0.89 ± 0.14
	Adjusted *R*^2^	0.84 ± 0.20
	*F* statistic of regression	106 ± 184
	*p*-value of regression	0.048 ± 0.085
(Log *K*^+^, Log *H*^+^)	*R*^2^	0.86 ± 0.18
	Adjusted *R*^2^	0.81 ± 0.24
	*F* statistic of regression	290 ± 908
	*p*-value of regression	0.053 ± 0.089
(*K*^−^, σ^−^)	*R*^2^	0.88 ± 0.16
	Adjusted *R*^2^	0.81 ± 0.27
	*F* statistic of regression	64 ± 95
	*p*-value of regression	0.062 ± 0.12
(*K*^+^, σ^+^)	*R*^2^	0.87 ± 0.19
	Adjusted *R*^2^	0.79 ± 0.32
	*F* statistic of regression	887 ± 4,397
	*p*-value of regression	0.075 ± 0.16

### Pooled classical and popular music analysis

#### Group-level (*K, H*), (*K*, σ), and (*H*^+^, *H*^−^) analyses

**Figure 10:**
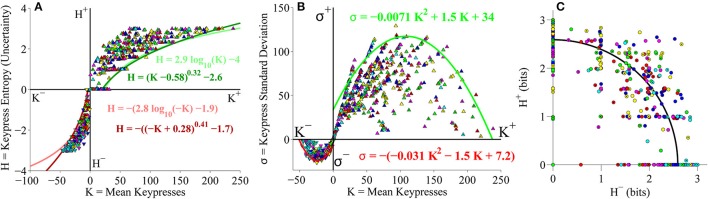
**Group relative preference results for pooled Classical and Popular music stimulus sets in the primary experiment. (A)** Approach and avoidance boundary envelopes for (*K, H*) data were fit by logarithmic functions (light red/green traces) and power-law functions with offset (dark red/green traces). Symbols depict (*K, H*) data pairs for the six Classical and six Popular music categories across all subjects in the cohort. Popular music categories are indicated by a black dot in the center of the symbols. **(B)** Boundary envelopes for the group (*K*, σ) data were fit with quadratic functions (green/red traces). Symbols depict (*K*, σ) data pairs for all Classical and Popular music categories and all subjects. **(C)** Trade-off plot showing group (*H*^+^, *H*^−^) data. Symbols indicate (*H*^+^, *H*^−^) data pairs for each individual Classical and Popular music category within individual subjects. Black line indicates radial fit computed across all subjects and music categories, such that $r=\sqrt{{\left(H^{+}\right)}^{2}+{\left(H^{-}\right)}^{2}}$.

As a final analysis, we took the second (larger) cohort of subjects from the primary experiment and pooled data together across both Classical and Popular music stimulus sets, to perform a relative preference analysis for subjects keypressing to a total of 12 music categories spanning different musical genres (six Classical music categories and six Popular music categories). Results of this pooled analysis corroborated the results observed for the pilot experiment as well as the analyses conducted separately on Classical and Popular music categories. Figure 10 displays the results of the group-level analysis across genres. Figure 10A depicts the group (*K, H*) value functions; boundary envelopes to approach and avoidance data were well fit by either logarithmic functions (light traces) or power-law functions with offsets (dark traces). Boundary envelopes to group (*K*, σ) data were well fit by quadratic functions (Figure 10B), and group (*H*^+^*, H*^−^) trade-off data exhibited a radial distribution with a mean radial distance of 2.59 bits (Figure 10C).

#### Individual subject (*K, H*), (*K*, σ), and (*H*^+^, *H*^−^) analyses

**Figure 11:**
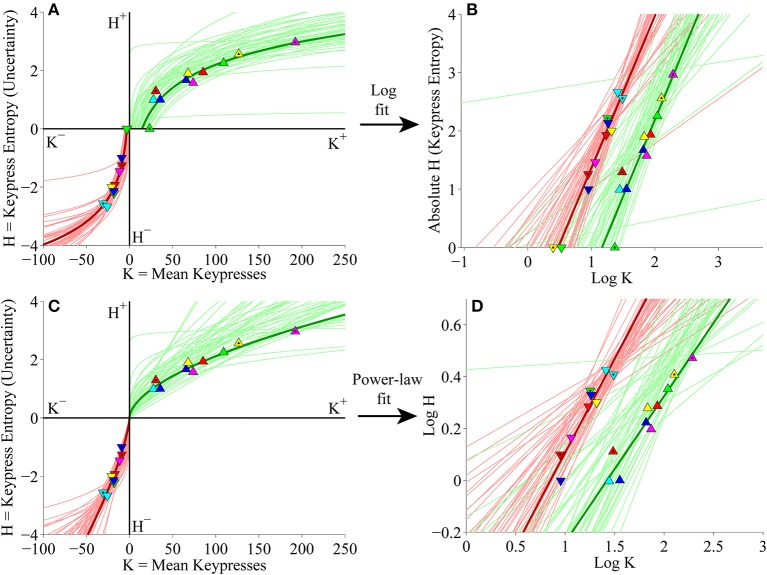
**Individual (*K*, *H*) value functions and (*K*, *H*) regressions for pooled Classical and Popular music stimulus sets in the primary experiment. (A)** Logarithmic fits to (*K, H*) data within individual subjects. Symbols indicate (*K, H*) values within individual Classical and Popular music categories for approach and avoidance keypressing in a representative subject. Popular music categories are indicated by a black fill in the center of the symbols. Dark green/red traces indicate log fits for approach/avoidance keypressing in a representative subject; light green/red traces indicate log fits for remaining subjects of the cohort. **(B)** Semilog plot illustrating linear regression of *H* vs. log_10_ *K* for approach (green) and avoidance (red) data in a representative subject (dark traces) and remaining subjects in cohort (light traces). **(C)** Simple power-law fits to (*K, H*) data within individual subjects, including data from a representative subject. **(D)** Log-log plot illustrating linear regression of log_10_ *H* vs. log_10_ *K* for the approach and avoidance data in individual subjects, including data from a representative subject.

At the level of individual subjects, value functions for (*K, H*) data across the 12 pooled music categories were well fit with logarithmic (Figure 11A) or power-law (Figure 11C) functions.

**Figure 12:**
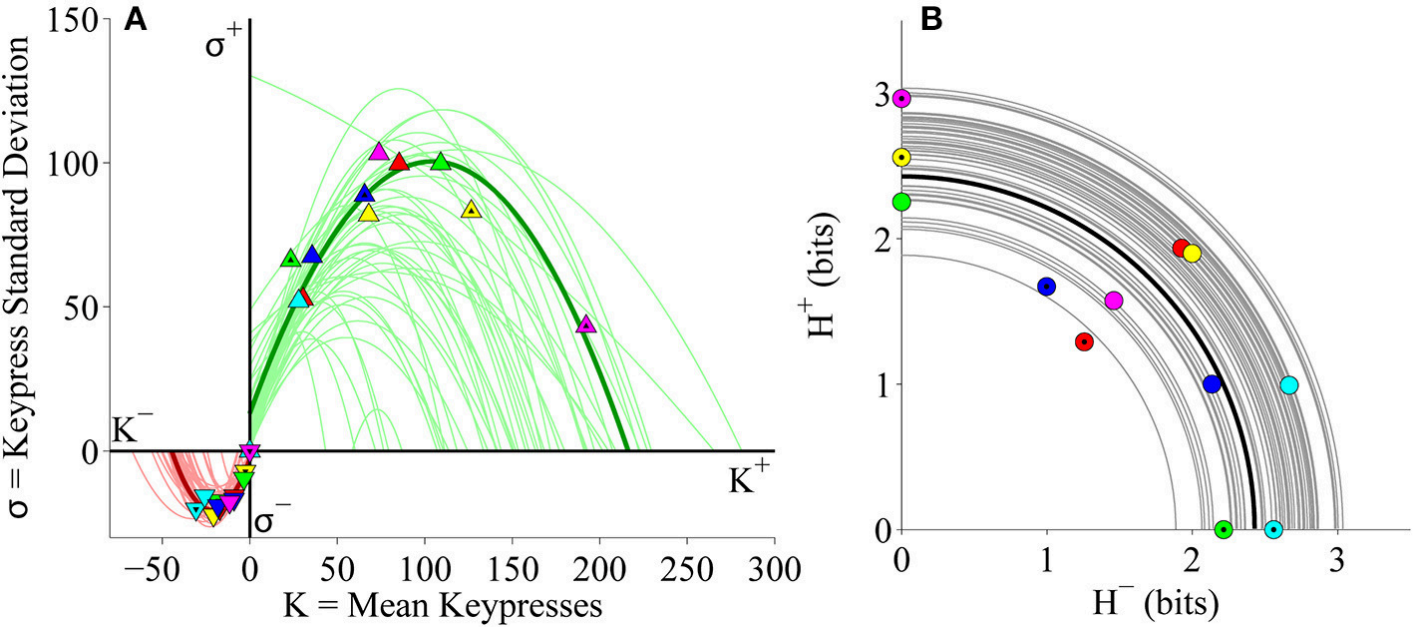
**Limit and trade-off plots for individual subjects from pooled Classical and Popular music stimulus sets in the primary experiment. (A)** Limit functions show the relationship between mean keypressing (*K*) and keypress standard deviation (σ) across Classical and Popular music categories within individual subjects, highlighting the representative subject (symbols, dark traces). Popular music categories are distinguished by a black fill in the center of the symbols. Limit functions for approach and avoidance keypressing are quadratic fits (σ = *a K*^2^ + *b K* + *c*). **(B)** Trade-off plot illustrates the relationship between avoidance (*H*^−^) and approach (*H*^+^) keypress entropy across Classical and Popular music categories, within individual subjects. Radial fits were computed for each subject such that $r\;=\;\sqrt{{\left(H^{+}\right)}^{2}+{\left(H^{-}\right)}^{2}}$. For both **(A,B)**, symbols and dark trace indicate data and fit for a representative subject; radial fits for remaining subjects are indicated by light traces.

The simple linear regressions of *H* against log_10_ *K* and log_10_ *H* against log_10_ *K* used to estimate the logarithmic and power-law fits (respectively) are displayed in Figures 11B,D, highlighting the results for the representative subject. Additionally, individual subjects exhibited (*K*, σ) limit functions conforming to quadratic functions (Figure 12A), and their individual (*H*^+^*, H*^−^) data were radially distributed around the origin of the trade-off plot, as shown in Figure 12B. Table 4 presents the goodness of fit estimates for fits to individual subjects' (*K, H*) and (*K*, σ) data for the pooled Classical and Popular music analysis.

**Table 4:** **Mean goodness of fit statistics for value and limit functions for music categories for pooled Classical and Popular music stimulus sets (from the primary experiment)**.

**Variables**	**Parameter**	**Mean ± SD for parameters**
(Log *K*^−^, *H*^−^)	*R*^2^	0.88 ± 0.14
	Adjusted *R*^2^	0.86 ± 0.18
	*F* statistic of regression	679 ± 1,623
	*p*-value of regression	0.010 ± 0.052
(Log *K*^+^, *H*^+^)	*R*^2^	0.82 ± 0.20
	Adjusted *R*^2^	0.80 ± 0.23
	*F* statistic of regression	302 ± 818
	*p*-value of regression	0.020 ± 0.091
(Log *K*^−^, Log *H*^−^)	*R*^2^	0.86 ± 0.13
	Adjusted *R*^2^	0.85 ± 0.15
	*F* statistic of regression	120 ± 128
	*p*-value of regression	0.0049 ± 0.016
(Log *K*^+^, Log *H*^+^)	*R*^2^	0.80 ± 0.20
	Adjusted *R*^2^	0.77 ± 0.23
	*F* statistic of regression	84 ± 88
	*p*-value of regression	0.025 ± 0.071
(*K*^−^, σ^−^)	*R*^2^	0.86 ± 0.12
	Adjusted *R*^2^	0.83 ± 0.15
	*F* statistic of regression	92 ± 205
	*p*-value of regression	0.0035 ± 0.012
(*K*^+^, σ^+^)	*R*^2^	0.82 ± 0.22
	Adjusted *R*^2^	0.78 ± 0.26
	*F* statistic of regression	74 ± 96
	*p*-value of regression	0.033 ± 0.12

### Goodness of fits for the (*K, H*) value function

Given issues in the literature between use of a logarithmic vs. a power-law framework for the Weber-Fechner Law (Fechner, 1860; Stevens, 1961), we further evaluated the issue of whether the (*K, H*) value functions are better fit by logarithmic or power-law functions. Our prior work has shown that both frameworks appear to work well within the range of interpretable human keypressing (Breiter and Kim, 2008; Kim et al., 2010; Lee et al., 2015). In this manuscript, we addressed this question by comparing the *R*^2^ values for the approach and avoidance curves between the logarithmic and power-law fits, in order to see whether one of the functions had significantly higher goodness of fit values. Considering results for approach and avoidance curves across four datasets (pilot experiment, Classical stimulus set in the primary experiment, Popular stimulus set in the primary experiment, pooled Classical and Popular analysis from the primary experiment) resulted in eight comparisons, requiring a *p*-value of 0.05/8 = 0.0063 after Bonferroni correction. None of the differences in *R*^2^ values between logarithmic and power-law fits to the (*K, H*) value functions approached this level of significance. For the pilot Classical music dataset, the fits to the avoidance curves had *R*^2^-values (±SD) of 0.91 ± 0.11 and 0.85 ± 0.22 for log and power-law fits (see Table 1), respectively, and were not statistically different (two-sample paired *t*-test, *p* = 0.19). Fits to the approach curves had *R*^2^-values of 0.85 ± 0.17 and 0.76 ± 0.29 for log and power-law fits (see Table 1), again not statistically different (*t*-test, *p* = 0.27). Likewise, there were no differences in *R*^2^-values of the fits to the (*K, H*) data for the second (primary) Classical music dataset (avoidance curves, log: 0.88 ± 0.14, power: 0.86 ± 0.19, *p* = 0.31; approach curves, log: 0.84 ± 0.20, power: 0.84 ± 0.22, *p* = 0.64; see Table 2), the Popular music dataset (avoidance curves, log: 0.90 ± 0.12, power: 0.89 ± 0.14, *p* = 0.76; approach curves, log: 0.88 ± 0.19, power: 0.86 ± 0.18, *p* = 0.93; see Table 3), or for the pooled Classical and Popular music dataset (avoidance curves, log: 0.88 ± 0.14, power: 0.86 ± 0.13, *p* = 0.07; approach curves, log: 0.82 ± 0.20, power: 0.80 ± 0.20, *p* = 0.03; see Table 4). Overall, average *R*^2^-values ranged from 0.82 to 0.91 and 0.76 to 0.89 for the log and power-law fits, respectively. Thus, both logarithmic and power-law functions produced consistently good fits to the (*K, H*) data, and we remain agnostic as to which form may be the preferred method for approximating the value function.

### Goodness of fits for the (*K*, σ) value function

Across analyses, average *R*^2^-values ranged from 0.82 to 0.88 for the quadratic fits to the (*K*, σ) value function (see Tables 1–4).

### Loss aversion measures

In the behavioral finance and neuroeconomics literature, loss aversion is defined as the overweighting of losses relative to gains, or, more technically, the slope (i.e., first derivative) of the avoidance curve of the value function divided by the slope of the approach curve (Kahneman and Tversky, 1979; Köbberling and Wakker, 2005; Schmidt and Zank, 2005). For this study, we compared a measure similar to the classic definition of loss aversion as defined under prospect theory (Lee et al., 2015), which we refer to as loss aversion going forward. Namely, we computed loss aversion from the relative slopes of the approach and avoidance curves fit to subjects' (*K, H*) value function data. For our data, loss aversion for the approach and avoidance curves was computed at the point where *K* = 25 keypresses. Kahneman and Tversky originally reported a value of 2.25 for loss aversion (Kahneman and Tversky, 1979), indicating that humans are more than twice as averse to losses as they are drawn to gains. The values we obtained for loss aversion in the (*K, H*) value functions of individual subjects keypressing to music stimuli overlapped this value in many cases. Table 5 provides the average loss aversion values computed across subjects for both the pilot experiment (Classical music only) and the two stimulus sets (Classical and Popular music) in the primary experiment. For the pilot Classical music dataset, logarithmic fits yielded an average loss aversion (±SD) of 1.13 ± 0.33, while power-law fits yielded average loss aversion of 3.26 ± 1.24. Thus, power-law fits overlapped the classical value reported by Kahneman and Tversky, while the values obtained from logarithmic fits were lower than this value. For the primary Classical music experiment, log fits produced average loss aversion of 1.38 ± 1.12, vs. 2.36 ± 3.47 for power-law fits, overlapping the classical value in both cases. For Popular music stimuli, loss aversion values were 0.97 ± 0.38 for log fits and 5.27 ± 13.48 for power-law fits. Finally, for the pooled Classical and Popular music analysis (from the primary experiment), loss aversion values were 1.26 ± 1.59 for log fits and 2.27 ± 0.82 for power-law fits, again overlapping the classical value of 2.25 in both cases.

**Table 5:** **Loss aversion values observed in RPT analyses for (*K, H*) value functions fit with logarithmic and power-law functions**.

**Stimulus set (Experiment)**	**Regression**	**Fit type**	**Loss aversion ± SD**
Classical music (pilot)	(log_10_ *K, H*)	Log	1.13 ± 0.33
	(log_10_ *K*, log_10_ *H*)	Power	3.26 ± 1.24
Classical music (primary)	(log_10_ *K, H*)	Log	1.38 ± 1.12
	(log_10_ *K*, log_10_ *H*)	Power	2.36 ± 3.47
Popular music (primary)	(log_10_ *K, H*)	Log	0.97 ± 0.38
	(log_10_ *K*, log_10_ *H*)	Power	5.27 ± 13.48
Pooled Classical and Popular music (primary)	(log_10_ *K, H*)	Log	1.26 ± 1.59
	(log_10_ *K*, log_10_ *H*)	Power	2.27 ± 0.82

### Subjectivity of preference

**Figure 13:**
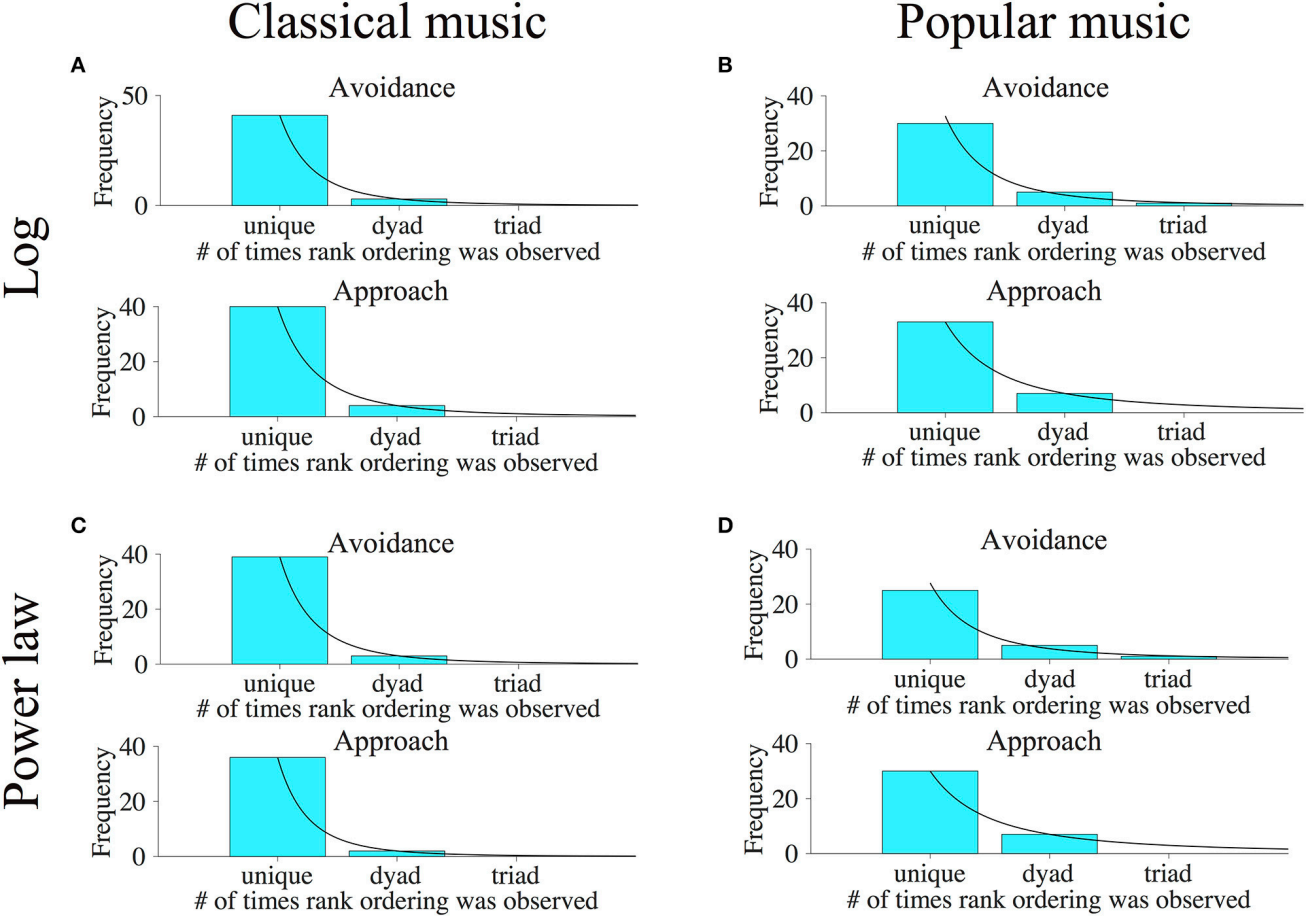
**Results of relative ordering analyses for primary Classical and Popular music experiments**. To determine the individuality of music preferences across subjects, we determined the rank ordering of music categories along the (*K, H*) value functions of individual subjects for both approach and avoidance keypressing behavior. Histograms were then generated that indicate the number of subjects having rank order combinations observed once (i.e., unique orderings), twice (orderings in two people or dyads), or more. The horizontal axes indicate the number of subjects who have a specific rank ordering in common. For instance, a value of one indicates rank orderings observed in only a single subject, a value of two indicates rank orderings observed across two subjects, etc. The vertical axes indicate the number of subjects in which a given rank order incidence was observed. Rank orderings observed in large numbers of subjects (i.e., large value on horizontal axis) indicate stereotyped patterns of music preference across subjects, while large numbers of rank orderings observed in only one or a small number of subjects (i.e., small value on horizontal axis) reflect individualized patterns of preference. **(A,B)** indicate the number of subjects having unique vs. not unique orderings of music categories using logarithmic (*K, H*) value functions for avoidance (top panels) and approach (bottom panels) keypressing to Classical and Popular music categories, respectively. **(C,D)** show similar frequency histograms of rank orderings for the Classical and Popular music categories, but were determined using the power-law fits to (*K, H*) value functions. Black lines indicate power-law fits to the rank order frequency distributions for **(A–D)** when there are three points to be fitted in the histograms; in histograms without three or more points for fitting, these black lines represent schema of a potential fit.

Finally, we wished to consider the extent to which approach/avoidance preferences were unique to individual subjects. If subjects all had similar preferences to approach or avoid the various categories of music stimuli, this would argue that preferences on the music keypress paradigm are not subjective, but are more common across all subjects. On the other hand, if individual subjects had unique patterns of approach/avoidance across music categories, this would argue that preference truly is subjective (i.e., individualized). To address this question, we examined the relative rank orderings of Classical and Popular music categories along the (*K, H*) value function for each individual, considering both logarithmic and power-law fits, using methods developed to assess relative preference logic in Kim et al. (2010) and described in Methods. Figure 13 shows frequency histograms that indicate the numbers of subjects who share rank orderings in common, when all categories of music had (*K, H*) values [thus individuals with 5 or less categories on the (*K, H*) value function were not included in the histograms].

The horizontal axes in these histograms specify the number of subjects who share a particular rank ordering in common. For instance, a value of one on the horizontal axis indicates rank orderings observed in only a single subject, a value of two indicates number of subjects having rank orderings common across two subjects (a dyad), a value of three indicates number of subjects with the same ordering in three subjects (a triad), etc. The vertical axis indicates the number of subjects observed with each type of rank ordering. (For instance, a bar with a frequency of 40 centered on *x* = 1 indicates 40 subjects with completely unique rank orderings seen in no other subject.) Rank orderings observed in common across many subjects (i.e., large value on horizontal axis) indicate stereotyped patterns of music preference, while rank orderings observed in only one or a small number of subjects (i.e., small value on horizontal axis) reflect individualized patterns of preference.

For the pilot Classical music phase, we found that all subjects with valid avoidance curve fits had completely unique rank preference orderings of avoidance to music categories for both power-law (15 of 15 valid subjects) and log (16 of 16 valid subjects) fits. For approach behavior, 14 of 16 valid subjects had unique preference orderings for the log fit, and 13 of 15 valid subjects had unique preference orderings for the power-law fit.

The results for the primary Classical and Popular music experiments are depicted graphically in Figure 13. Rank ordering analysis derived from logarithmic fits to the value functions produced the following results: For Classical music, avoidance rank orderings were unique in 41 of 47 subjects, whereas 3 pairs of people shared common rank orders (3 dyads). This produced a split between 87% unique and 13% shared rank orderings of preference. The approach rank orderings for Classical music were unique in 40 of 48 subjects, whereas 4 pairs of subjects shared common rank orders (4 dyads). This resulted in a split between 83% unique and 17% shared rank orderings of preference. For Popular music, avoidance rank orderings were unique in 30 of 43 subjects, whereas there were 5 pairs of subjects who shared common rank orderings (5 dyads), and 3 subjects shared a single rank order (1 triad). This produced a split between 70% unique and 30% shared rank orderings of preference. The approach rank orderings for Popular music were unique in 33 of 47 subjects, whereas 7 pairs of subjects shared common rank orders (7 dyads). This resulted in a split between 70% unique and 30% shared rank orderings of preference. Notably, we also observed that in all analyses, the frequency distributions of rank orderings could be well approximated by power-law functions where there were three data points (see black lines in Figure 13). In Figure 13, where a histogram has fewer than three points for fitting, these black lines represent a “perfect” or idealized fit.

For the rank orderings in the primary experiments derived from power-law fits to the value functions shown in Figure 13, we observed the following. For Classical music, avoidance rank orderings were unique in 39 of 45 subjects, whereas 3 pairs of subjects shared common rank orders (3 dyads). This produced a split between 87% unique and 13% shared rank orderings of preference. The approach rank orderings for Classical music were unique in 36 of 40 subjects, whereas 2 pairs of subjects shared common rank orders (2 dyads). This resulted in a split between 90% unique and 10% shared rank orderings of preference. For Popular music, avoidance rank orderings were unique in 25 of 38 subjects, whereas there were 5 pairs of subjects who shared common rank orders (5 dyads), and another 3 subjects who shared a single rank order (1 triad). This produced a split between 66% unique and 34% shared rank orderings of preference. The approach rank orderings for Popular music were unique in 30 of 44 subjects, whereas 7 pairs of subjects shared common preference orderings (7 dyads), resulting in a split between 68% unique and 32% shared rank orderings of preference.

Finally, for the pooled Classical and Popular music analysis, 48 and 47 of 49 subjects had valid fits computed for the (*K, H*) avoidance curve for logarithmic and power-law fits, respectively, and were used for analysis of preference rank ordering. For approach behavior, 49 and 48 of 49 subjects had valid curve fits computed for log and power-law fits. Among these valid subjects, every single subject had a unique preference rank ordering when categories were pooled across both classical and popular music stimuli.

Across primary experiments and stimulus sets, the majority of subjects had unique relative orderings of music categories along the (*K, H*) value function: ~66–90% unique (average of 78%) vs. 10–34% shared (average of 22% shared). Our findings thus argue that keypress-based musical preferences are, to a considerable extent, unique to the individual.

As a secondary analysis to the analysis of preference subjectivity, we sought to test if a history of music lessons or self-described music expertise were associated with the general 78% unique vs. 22% shared distribution of preferences. For this analysis, there were not enough data points in the 22% shared group for a valid ANOVA by the Fit type (log or power law), by Music task (Classical us. Popular music), or by Curve Type (approach or avoidance).

## Discussion

This study observed patterns of approach and avoidance, in two separate cohorts with two genres of music stimuli, that showed individual variability yet were lawful. Specifically: (1) Dynamic auditory stimuli produced RPT functions similar to the three functions reported from static visual stimuli and schematized in Figures 1A–C. (2) RPT functions from music stimuli, like static visual stimuli, reflected the value function in prospect theory and the construct of decision utility from portfolio theory. Metrics derived from these graphs, notably loss aversion, were similar to what has been reported in the literature with visual stimuli for RPT and for prospect theory. (3) RPT functions, across group and individual data, showed a similarity in their mathematical fitting that is consistent with scaling across levels of organization. (4) Approximately 78% of subjects showed unique patterns of category preference not shared by more than one other subject, yet a minority of 22% of subjects had exactly the same ordering of music categories, raising the hypothesis of a Pareto distribution defining a power law relationship between shared and unique preference orders, potentially related to the power law fits observed between {*K, H*} variables in this study (Barry, 1983; Hardy, 2010).

The demonstration that relative preference variables exhibited the same patterns on a keypress task involving time-varying, auditory stimuli as observed with static visual stimuli raises the hypothesis that the relationships observed among these variables are likely domain-general and occur for a wide range of stimuli in reward-aversion processing. Graphs from music data showed *R*^2^ consistently greater than 0.8, like the studies with visual stimuli. Our “loss aversion” findings support this in that our pooled Classical/Popular music data with the power-law fit (parallel to that used by Kahneman and Tversky, 1979) produced a loss aversion metric that almost exactly matched the classic literature value (2.27 for our data vs. 2.25 reported in literature; Tversky and Kahneman, 1992). Notably, the RPT loss aversion metrics observed with music stimuli were similar to those observed with visual stimuli and to those from a prospect theory paradigm in the same experimental subjects (Lee et al., 2015). These findings with music stimuli extend the potential use of this keypress task for behavioral neuroscience beyond applications with static stimuli or visual stimuli, to use with time varying stimuli and auditory stimuli, and allow a lawful description of the resulting behavior while preserving a broad potential diversity of individual preference behavior.

Richard Feynman considered scaling to be a fundamental feature of lawfulness (Feynman, 1965). The relationships among relative preference variables we observed—the (*K, H*) value functions, (*K*, σ) limit functions, and (*H*^+^, *H*^−^) trade-off plots—appeared to scale, with consistent mathematical descriptions between individual subjects and the group level. Furthermore, scale invariance was apparent given the high *R*^2^-values obtained for either power-law or logarithmic fits to the (*K, H*) data. With simple power-law fitting, scale invariance was verified by performing linear regression following log transformation of both the *K* and *H* axes. The resulting fits characteristically demonstrated asymptotic behavior (0 < *a* < 1, given *H = b K*^*a*^), which implies that substantial changes in the input variable (*K*) produce only minor changes in the output (*H*). It is worth noting that such compression of the range of possible outputs is a hallmark of biological and other complex systems (Gisiger, 2001; Freyer et al., 2012). The same asymptotic behavior was characteristic of the logarithmic fits to the (*K, H*) data, with the difference that in this case the fits were obtained by performing linear regression of *H* against *K* after log transformation of *K* alone. Connections between layers of organization in a complex system specify the information that one layer has about other layers (Szostak, 2003). Such connections between levels, as exhibited in the preserved graphical relationships between relative preference variables across individual and group levels, suggest that the principles underlying organization at one scale are preserved at the other (Sutton and Breiter, 1994; Kim et al., 2010). Scaling means that a particular description of a behavior or an object does not change if the scale of time, weight, height or other parameters are dilated or contracted by a common factor. The issue of scaling (or scale invariance) has become a fundamental construct in neuroscience (see Braeutigam commentary in Frontiers of Neuroscience, 2017), particularly as neuroscience seeks to connect and differentiate measurements made as distinct levels of spatio-temporal organization, such as between behavior, distributed ensembles of circuits, micro-circuits, cells, etc. (Bohland et al., 2009).

The observation of scaling and lawfulness in this music data occurred in parallel with response probability distributions showing the majority of subjects had unique preference orderings. Namely, the majority of individuals in this experiment had a broad diversity of preference toward categories of music, yet these profiles could all be consistently fit with RPT functions. Frequency analysis of preference rank order (i.e., Figure 13) could be fit with power-law functions and logarithmic fits, consistent with the use of either function for fitting the keypress value function (Figures 3–5, 7, 8, 10, 11). These observations support the contention that the RPT graphs of music data are lawful (Feynman, 1965), yet allow for individual variability in the pattern of preference, arguing that RPT allows for the diversity of human preference while providing structure for it.

It is intriguing that the distribution of unique to shared responses across subjects was ~78–22%, showing strong inhomogeneity in this joint ratio, akin to a Pareto distribution (Barry, 1983; Hardy, 2010). A Pareto distribution is a significantly skewed probability distribution that produces power-law fits tending to scale across levels of spatio-temporal organization, like those observed in this study. There are explicit procedures for testing if data reflects an underlying Pareto distribution, but much larger data sets than ours are needed for such testing, and for ruling out that our 78–22% split is not just a statistical anomaly akin to the birthdate problem—in a room of people, what are the odds you share the same birthday? With 39 instantiations of a 6 digit random number (given only numbers 1–6 and non-replacement), there is a 65% probability that a number will be repeated. Accordingly, at this time, the 78–22% split between unique and shared preference orderings in subjects can only be interpreted as supporting a hypothesis of a Pareto distribution underlying musical preference, and needs at least another 2–4 cohorts to be tested, with cohort sizes potentially into the hundreds if not thousands of subjects. If one were to produce such a study, one would explicitly test if the alpha-exponent of the decay function fit to the response probability distribution (e.g., black curved lines in Figure 13) either define a power law relationship between shared and unique preference orders, or is potentially associated with the exponent for the power-law fit of the (*K, H*) value function (e.g., Figures 3–5, 7, 8, 10, 11).

Beyond such testing, additional or intermediate variables may also exist which moderate or mediate relations between rank orderings of preference, and the lawful patterns between {*K, H*, σ} variables we observed [i.e., reflect an interaction effect (moderation) or an additive effect (mediation), respectively]. For instance, it is possible that variables of music familiarity and music training may have a relationship to the response probability function, something we did not have the cohort size to test (see last paragraph in *Subjectivity of preference* in Results). Also, prior RPT work has shown that hedonic deficit states of hunger/satiation shift the position of categorical preferences on the (*K, H*) value functions, (*K*, σ) limit functions, and (*H*^+^, *H*^−^) trade-off plots of individuals, but do not alter the mathematical fits of these functions even when these studies are done more than a week apart (Kim et al., 2010). Such work argues that RPT curves may provide a method for quantifying familiarity and musicianship effects on preference. People generally like a song more after they have heard it a few times, but many repetitions can reduce this preference, suggesting an upside down U curve between exposure (x-axis) and preference (y-axis) (Bornstein, 1989). It is possible that the (*K*, σ) limit function may provide a framework for mapping familiarity effects given its similarity to an upside down U curve, if exposure is added as a z-axis to the *K* and σ axes.

Consistent with prior publications using visual stimuli (e.g., Breiter and Kim, 2008; Perlis et al., 2008; Gasic et al., 2009; Kim et al., 2010; Lee et al., 2015; Viswanathan et al., 2015, 2017), the current music data demonstrate offsets from the origin of the value function graph where the positive and negative value functions intersect the *x*-axis. Modeling that incorporates such offsets (i.e., logarithmic fits with additive offsets, as opposed to simple power-law fits) consistently shows higher *R*^2^ results, suggesting the offsets are not artifacts but reflect an important aspect of the underlying system. Such offsets have ready analogies to existing behaviors in decision-making during uncertainty. Namely, the positive offset can be analogized to an “ante” in poker, where a player puts a baseline bet into the kitty of existing bets. The negative offset can be analogized as taking a hedge against a potential loss, or investing in insurance to cover uncertain negative outcomes. Prior work with prospect theory (Kahneman and Tversky, 1979) and matching from operant conditioning (Herrnstein, 1961) appear to disallow such offsets; further work is needed to better understand the role of these offsets in reward/aversion behavior as well as their physiological basis.

The observation of recurrent patterns among approach/avoidance variables to music stimuli has potential implications beyond behavioral neuroscience to the neuroscience of music preference and recommendation engines. Keypressing is readily translated into the duration of approach and avoidance, and exposure duration has been shown to follow an RPT framework (Breiter and Kim, 2008; Kim et al., 2010). The duration of time a user listens to a music track before skipping to the next song (e.g., data collected by iTunes) could be used as a surrogate measure of preference intensity, instead of a keypress measure of preference. This would permit online music providers to monitor the preferences of their users to all accessed songs in order to suggest similar music for which the user is likely to have a strong preference.

There are also salient implications of the current findings for music neuroscience. Important research has indicated that preference ratings, such as Likert scale approaches (Likert, 1932) to quantifying music preferences are associated with reward circuitry activation (e.g., Blood and Zatorre, 2001; Salimpoor et al., 2011; Trost et al., 2012), and that physiological biometrics of preference can be both associated with preference ratings (e.g., Blood and Zatorre, 2001; Grewe et al., 2007; Guhn et al., 2007; Salimpoor et al., 2009; Laeng et al., 2016) and with activation in reward circuitry connected with music responses (e.g., Blood and Zatorre, 2001; Osuch et al., 2009; Pereira et al., 2011; Trost et al., 2012; Salimpoor et al., 2013). The current results point to a number of direct neuroimaging and physiology applications that can add to these ongoing neuroscience efforts. First, the RPT framework produces a rank ordering of preferences that fulfills criteria for preference logic (Hansson and Grune-Yanoff, 2009), and is done within a framework of lawful behavior (Kim et al., 2010). Such a framework provides a complementary approach to Likert scales, particularly excelling at relative ordering of preference within in individual. Second, the pattern variables used in RPT, such as the Shannon entropy measure *H*, can be directly studied against brain activation, and to our knowledge, have only been used once to date with regard to the patterns in human judgment and decision-making (e.g., Viswanathan et al., 2015). *H* describes the uncertainty or information contained within a pattern of judgments or choices, and as such provides a framework by which to study how the brain organizes preference information. It also allows an investigator to assess effects of divergence in decision making, to understand how the individual can have large degrees of freedom for any particular judgment or choice, but still be constrained by the balance between a set of approach decisions and a set of avoidance decisions. The (*H*^+^, *H*^−^) tradeoff relationship in particular provides a mechanism for having lawful determinism over bundles of choices, along with unconstrained choice at any particular decision point, and as such, provides a fundamental construct for studying the balance between the two. Third, the fitting parameters of the RPT value function, saturation function, and tradeoff function can all be used in linear and non-linear regression analyses, or support vector machine analyses, to identify brain regions involved with aligning the rank orders of preference, along with their dynamic shifting for state-based or contextual factors. For instance, we currently have minimal neuroscience understanding of how core constructs such as loss aversion may change over time, or relate to dynamic stimuli such as music (e.g., see Viswanathan et al., 2015). Nor do we have a strong neuroscience basis for how rank preference ordering may change over the course of circadian cycles, or for contextual factors that make one type of music potentially something the individual does not want to appear to enjoy (or vice versa). These three approaches to using RPT with neuroimaging and physiology suggest a number of opportunities for furthering music neuroscience.

In summary, the present study used music stimuli to identify discrete, recurrent and scalable patterns for approach/avoidance behavior that were consistent with patterns observed with static visual stimuli. These findings suggest that RPT patterns may be general across sensory domains and extend to dynamic, time-varying stimuli. Individual preference was quite heterogeneous across subjects, yet most subjects showed strong RPT curve fits. This argues that musical preferences can be quite individualistic, showing variance along functions that are lawful yet provide a scaffold for this variance. The ability to quantify individual differences in this rigorous manner may advance the precision with which we are able to predict individual responses to music, both from a behavioral standpoint and when mapping to underlying biology. These observations may also have implications for a number of domains ranging from behavioral and music neuroscience to improvement of music recommendation systems.

## Author contributions

JC, MB, AJB, and HB developed the study concept. BK, ML, EM, MB, AJB, and HB produced the study design, which was revised by SL, JS, JB, AEB, and JC. Testing and data collection were performed by SL, BK, ML, and VM while SL, JS, BK, and ML performed initial data analysis with guidance of JC, AJB, and HB. Interpretation was performed by SL, JS, BK, AJB, and HB with input from EM, AEB, KO, MB, and JC. Initial full draft of the paper was by SL, JS, AJB, and HB. Significant revisions were provided by BK, EM, JB, MV, KO, JC, and MB. All authors approved the final version of the paper for submission.

## Funding

Primary support for HB and team was provided by the Warren Wright Adolescent Center at Northwestern Memorial Hospital and Northwestern University, Chicago, IL. Support was also provided by a grant to AB (#052368) from NINDS, Washington, DC, and a grant from the Dystonia Medical Research Foundation to AB. The funders had no role in study design, data collection and analysis, decision to publish, or preparation of the manuscript.

### Conflict of interest statement

The authors declare that the research was conducted in the absence of any commercial or financial relationships that could be construed as a potential conflict of interest.
