# Supplementary material for: Keypress-Based Musical Preference Is Both Individual and Lawful
Source: Front Neurosci. 2017 May 2;11:136. doi: 10.3389/fnins.2017.00136 (PMC5412065; doi:10.3389/fnins.2017.00136)
Supplement: Supplementary file 1 [file DataSheet1.docx]

Supplementary Material

Keypress-based musical preference is both individual and lawful

**Sherri L. Livengood**^1,2,†^, **John P.** **Sheppard JP**^1,2,3†^, **Byoung W.** **Kim**^1,2,4,†^, **Edward C.** **Malthouse**^2,5,‡^, **Janet E. Bourne**^2,6,‡^, **Anne E.** **Barlow**^2,7,‡^, **Myung J. Lee**^1,2,4,‡^, **Veronica Marin**^1,‡^**, Kailyn P. O’Connor**^1,‡^**,** **John G.** **Csernansky**^8,‡,#^, **Martin P.** **Block**^2,5,‡,#^, **Anne J.** **Blood**^2,4,9,10,11,†,#,^**^*^**, **and Hans C. Breiter**^1,2,4,9,10,†,#,^**^*^**

^1^Warren Wright Adolescent Center, Department of Psychiatry and Behavioral Sciences, Feinberg School of Medicine, Northwestern University, Chicago, IL, USA,

^2^Applied Neuromarketing Consortium, Medill, Kellogg, and Feinberg Schools, Northwestern University, Evanston, IL, USA,

^3^David Geffen School of Medicine, University of California-Los Angeles, Los Angeles, CA, USA

^4^Northwestern University and Massachusetts General Hospital Phenotype Genotype Project in Addiction and Mood Disorders, Boston, MA, USA,

^5^Medill Integrated Marketing Communications, Northwestern University, Evanston, IL, USA,

^6^Music Department, Bates College, Lewiston, ME, USA,

^7^KV 265, The Communication of Science through Art, Willow Springs, IL, USA,

^8^Department of Psychiatry and Behavioral Sciences, Feinberg School of Medicine, Northwestern University, Chicago, IL, USA,

^9^Mood and Motor Control Laboratory, Department of Psychiatry, Massachusetts General Hospital, Boston, MA, USA,

^10^Laboratory of Neuroimaging and Genetics, Department of Psychiatry, Massachusetts General Hospital, Boston, MA, USA,

^11^Department of Neurology, Massachusetts General Hospital, Boston, MA, USA,

†Joint first authorship, ‡Joint second authorship, # Joint senior authorship.

*** Correspondence:**For Relative Preference Theory and Analysis: Hans C. Breiter, MD, Warren Wright Adolescent Center, Department of Psychiatry and Behavioral Sciences, Northwestern University Feinberg School of Medicine, 710 N. Lake Shore Dr., Abbott Hall 1302, Chicago, IL, 60611, E-mail: [h-breiter@northwestern.edu](mailto:h-breiter@northwestern.edu)

For Music Reward and Music Theory: Anne J. Blood, PhD, Mood and Motor Control Laboratory, and Laboratory of Neuroimaging and Genetics, Departments of Psychiatry and Neurology, Massachusetts General Hospital, 120 2nd Avenue, Charlestown, MA 02129, E-mail: [ablood@nmr.mgh.harvard.edu](mailto:ablood@nmr.mgh.harvard.edu)

# Supplementary Information

**Materials and Methods**

***Additional material on participants***

After obtaining written informed consent, subjects were screened for inclusion and exclusion criteria. In both the pilot and primary experiments, subjects were normal controls that were determined to be free of any psychiatric, neurological, or medical issues per a psychiatrist-based Mini International Neuropsychiatric Interview (MINI; Lecrubier et al., 1997). Subjects in the pilot experiment had a mean educational history of 15.28 ± 2.49 years and a mean score of 98.88 ± 10.14 on the Wide Range Achievement Test, 4^th^ edition (WRAT 4; Wilkinson & Robertson, 2006). Subjects in the primary experiment had a mean educational history of 16.34 ± 2.30 years and a mean score of 109.76 ± 12.17 on the WRAT 4. There were no significant differences between men and women for age, education or WRAT 4 (pilot experiment: Age *p* = .44; Education *p* = .80; WRAT 4 *p* = .60; primary experiment: Age *p* = .73; Education *p* = .27; WRAT 4 *p* = 0.07). Race was determined by individual self-identification using a standardized form (Benson & Marano, 1998), and handedness via the Edinburgh Handedness Inventory (Oldfield, 1971). In the pilot experiment, subjects self-identified as 6 African-Americans, 7 Caucasians, 1 Asian, and 2 unknown, with 6 reporting as Hispanic and 10 as non-Hispanic. Fourteen of the 16 subjects were right-handed. In the primary experiment, subjects self-identified as 19 African-Americans, 18 Caucasians, 17 Asians, 1 Native American, and 7 unknown, with 9 self-reporting as Hispanic and 53 as non-Hispanic. Fifty-three of the 62 subjects in the primary experiment were right handed.

***Additional details on music keypress task***

Subject instructions for the experiment were presented on screen and were read aloud by the experimenter. The experiment began with the option to set the volume to a comfortable listening level for the remainder of the experiment, by pressing “1” to lower and “2” to increase volume (it could not be changed thereafter). This volume setting was followed by a practice round for the task, using three music excerpts that were not part of the stimulus set. Subjects were told they would hear a series of music excerpts, beginning with a preview to the excerpt, followed by a short silent period and then a longer version of the same excerpt. They were told they would have the option to increase, decrease or do nothing to change the listening time of the longer version of each excerpt. Subjects were directed to use their writing hand for all keypressing, and they were to press “N” and “M” alternatingly to increase or “Z” and “X” alternatingly to decrease the listening time of each music excerpt. They also had the option to do nothing and let the piece play for its fixed default amount of time. Subjects were informed that neither the total experiment time nor their payment would be affected by their keypress responses. Visually, feedback on the remaining length of listening time for each music excerpt was given via a red bar presented during the passive pre-listening stage, to indicate that no changes could be made, or a green bar during the active keypress listening stage, to indicate that the listening time could be increased or decreased. Following the practice round, to minimize the visual burden during the auditory task, only the timing feedback bars remained on the screen.

The relationship between the number of keypresses made by the subject to increase listening time (i.e., approach) or decrease listening time (i.e., avoid) of a musical excerpt followed previous methods (Aharon et al., 2001; Strauss et al., 2005), and utilized the following resistive function:

$t_{n}=\sum_{n=1}^{N} t_{n-1}+{(A-t_{n-1})}/J$, (1)

where *t­_n_* is the updated listening time achieved after the keypress, *t_n-1_­* is the allotted listening time prior to the keypress, *A* is equal to 0 seconds for avoidance keypresses reducing the listening time or 30 seconds for approach keypresses increasing the listening time, and *J* is a scaling constant equal to 40.

***Additional details on boundary envelope fitting for (K, H) value and (K, σ) limit functions***

We fit the group (***K***, ***H***) group-level boundary envelopes by parcellating the (***K, H***) space into six equally sized bins separated by ***H***, and 50 equally sized bins separated by ***K***. These bins defined a total of 300 rectangular quadrants in (***K, H***) space. For the center of each “***H***-bin,” we moved outward from the ***H =* 0** line until we reached the “***K***-bin” at which 95% of the data points within the current ***H***-bin had been encountered. The center of each such quadrant constituted a point in (***K***, ***H***) space; to these points, we fit either logarithmic functions or power-law functions with offsets to define the (***K***, ***H***) boundary envelopes. As seen in Figs. 2a, 3a, 6a, 9a, this method produced boundary envelopes closely matching the contour of the observed (***K***, ***H***) data.

For boundary envelope fitting to group (***K, σ***) data, we parcellated the (***K, σ***) space into 16 equally sized bins in terms of ***K***, and 40 equally sized bins in terms of ***H***. This process defined 640 equally sized rectangular quadrants in (***K, σ***) space. Then, for each ***K***-bin, we moved outward from the ***K =* 0** line until we reached the ***H-***bin at which 95% of the (***K, σ***) data had been encountered for the current ***K****-*bin. The center of such quadrants defined points in (***K, σ***) space representing the observed boundary of the data; to these points, we fit the quadratic boundary envelopes by using Matlab’s *polyfit* function. As shown in e.g. Fig. 2c, the quadratic boundary envelopes fit using this method closely followed the observed contours of the (***K, σ***) data.

**Results**

In the text that follows, specific subject exclusions are noted, and summarized in Tables S1-S5.

***Classical music pilot experiment***

*Individual subject (K, H) value functions*

All subjects demonstrated the same logarithmic shape for both approach and avoidance curves. In particular, we required that the scaling parameter ***a*** be positive (***a >* 0**), conforming to the shape of the group data (below); all 16 subjects met this criterion for both approach (positive) and avoidance (negative) curves. Subjects' value functions were also well fit by simple power-law functions of the form ***H = b K^a^*** (not shown). Fifteen of 16 subjects had sufficient data to fit the power-law function to the (negative) avoidance curve; one subject lacked sufficient data to fit the power-law function to the (positive) approach curve (Methods). For the power-law function, we required that the scaling parameter ***a*** fall within the range of **0 *< a <* 1**, as this shape was observed in the vast majority of subjects and conformed to the shape of the group (***K, H***) distributions (below). Twelve of the 15 surviving subjects met this criterion for the approach curves; the remaining three subjects' power-law fits for the approach curves were omitted from analysis. All surviving subjects met this criterion for the avoidance curves.

*Individual subject (K, σ) and (H^-^, H^+^) plots*

Because the vast majority of subjects displayed the same quadratic trend with an inverted U-shape (i.e. Fig. 2d), we required that the quadratic term ***a*** be negative (***a <* 0**). Fourteen of 16 subjects met this criterion for the approach curve, and 15 of 16 subjects met the criterion for the avoidance curves; in the remaining cases, the approach or avoidance curves not conforming to this criterion were omitted from Fig. 2d and excluded from analysis.

***Primary Classical music experiment***

*Individual subject (K, H) value functions*

For this analysis, 47 of 49 subjects had sufficient data to fit valid logarithmic functions to the avoidance curves, while 48 of 49 subjects had sufficient data to fit the approach curves. Among the surviving subjects, two had invalid logarithmic fits for the approach curve due to the sign of the logarithmic term (i.e., ***a >* 0**), and one subject had an invalid fit for the avoidance curve; these cases were excluded from analysis.

For the power-law fits, 40 of 49 subjects had sufficient data to compute a valid fit for the approach curve, while 45 of 49 subjects possessed sufficient data for the fits to the avoidance curves. Among the power-law fits, seven subjects had fits that were deemed invalid for the approach curve on the basis of the scaling parameter, and six subjects had invalid fits for the avoidance curve; these cases were omitted from Fig. 4c,d and excluded from further analysis.

*Individual subject (K, σ) and (H^-^, H^+^) analyses*

In this analysis, 48 of 49 subjects possessed sufficient data to compute the quadratic (***K, σ***) fit for the approach curves; 47 of 49 subjects had sufficient data to fit the avoidance curve. Among surviving subjects, five subjects possessed invalid fits for the approach curve (i.e., violated the condition that ***a < 0***), and four subjects possessed invalid fits for the avoidance curve.

***Pop music experiment***

*Individual subject (K, H) value functions*

For this dataset, 47 of 49 subjects had sufficient data to fit logarithmic functions to the approach data; 43 of 49 subjects had sufficient data to fit the avoidance data. Among subjects with sufficient data, all logarithmic fits were deemed valid and included in the analysis. For the power-law fits, 44 of 49 subjects possessed sufficient data to fit the approach curves, while 38 of 49 subjects possessed sufficient data for the avoidance curves. Of the surviving subjects, five subjects had power-law fits deemed invalid on the basis of the scaling parameter for the approach curves, and two subjects had invalid fits for the avoidance curves; these cases were omitted from analysis.

*Individual subject (K, σ) and (H^-^, H^+^) analyses*

For (***K, σ***) data, 47 of 49 subjects had sufficient data to perform quadratic fitting of the approach curve, and 43 of 49 subjects had sufficient data to fit the avoidance curve. Among surviving subjects, three subjects possessed invalid fits for the approach curve (i.e., ***a >* 0**), and one subject possessed an invalid fit for the avoidance curve; these cases were excluded from analysis.

***Pooled Classical and Popular music analysis***

*Individual subject (K, H), (K, σ), and (H^-^, H^+^) analyses*

All 49 subjects possessed valid approach fits for the logarithmic functions. For the avoidance fits, one subject had insufficient data to fit the logarithmic function; all remaining 48 subjects had valid avoidance fits. For the power-law fits, one subject possessed insufficient data to compute a fit to the approach curve, and two subjects had insufficient data to fit the avoidance curve. Among surviving subjects, two subjects had invalid power-law fits to the approach curve based on the scaling parameter, and two subjects also had invalid power-law fits to the avoidance curve; these cases were omitted from Fig. 10 and excluded from analysis.

All subjects had sufficient data to perform quadratic fits to the (***K, σ***) approach data, and 48 of 49 subjects had sufficient data to fit the avoidance data. Quadratic fits to the approach curves were omitted in two subjects due to violating the criterion that ***a <* 0**; the quadratic fits met this criterion in all surviving subjects for the avoidance curves, and so all were included in the analysis.

***Goodness of fits for the (K,* *σ) value function***

For the pilot Classical music dataset, the quadratic fits to the avoidance curves had *R^2^* values (± SD) of .85 ± .18 and approach curves had *R^2^* values of .82 ± .27 (see Table 1). In the primary experiment, the Classical music dataset had avoidance curves *R^2^* values of .88 ± .19, and approach curves had *R^2^* values of .82 ± .26; the Popular music dataset had avoidance curve *R^2^* values of .88 ± .16 and approach curve *R^2^* values of .87 ± .19 (see Tables 2-3). For the pooled Classical and Popular music dataset, the *R^2^* values for avoidance curves were .86 ± .12, and the approach curve *R^2^* values were .82 ± .22 (See Table 4). Overall, average *R^2^* values ranged from .82 - .88 for the quadratic fits to the ***(K,* *σ)*** value function.

# Supplementary Figures and Tables

Table S1: *Invalid datasets for pilot experiment analysis (N = 16)*

| RPT Graph | Fit type | Inclusion criteria | Approach curves | Avoidance curves | Total excluded curves |
| --- | --- | --- | --- | --- | --- |
| (*K*, *H*) | Boundary envelope | K > 0 at category level to produce H (Log of 0 is invalid) | 16/16 | 16/16 | 0/32 |
| (*K*, *H*) | Log fit (*H = a* log_10_*K + b*) | Data points ≥ 3 | 16/16 | 16/16 | 0/32 |
| (*K*, *H*) | Log fit (*H = a* log_10_*K + b*) | a> 0 | 16/16 | 16/16 | 0/32 |
| (*K*, *H*) | Power-law fit (*H = b K^a^*) | Data points ≥ 3 | 15/16 | 15/16 | 2/32 |
| (*K*, *H*) | Power-law fit (*H = b K^a^*) | 0 < a < 1 | 12/15 | 15/16 | 4/32 |
| (*K, σ*) | Boundary envelope | None | 16/16 | 16/16 | 0/32 |
| (*K, σ*) | Quadratic fit *(σ = a K^2^ + b K + c)* | Data points ≥ 4 | 16/16 | 16/16 | 0/32 |
| (*K, σ*) | Quadratic fit *(σ = a K^2^ + b K + c)* | a < 0 | 14/16 | 15/16 | 3/32 |

Table S2: *Invalid datasets for Classical stimuli analysis of primary data (N = 49)*

| RPT Graph | Fit type | Inclusion criteria | Approach curves | Avoidance curves | Total excluded curves |
| --- | --- | --- | --- | --- | --- |
| (*K*, *H*) | Boundary envelope | K > 0 at category level to produce H (Log of 0 is invalid) | 49/49 | 49/49 | 0/98 |
| (*K*, *H*) | Log fit (*H = a* log_10_*K + b*) | Data points ≥ 3 | 48/49 | 47/49 | 3/98 |
| (*K*, *H*) | Log fit (*H = a* log_10_*K + b*) | a > 0 | 46/48 | 46/47 | 6/98 |
| (*K*, *H*) | Power-law fit (*H = b K^a^*) | Data points ≥ 3 | 40/49 | 45/49 | 13/98 |
| (*K*, *H*) | Power-law fit (*H = b K^a^*) | 0 < a < 1 | 33/40 | 39/45 | 26/98 |
| (*K, σ*) | Boundary envelope | None | 49/49 | 49/49 | 0/98 |
| (*K, σ*) | Quadratic fit *(σ = a K^2^ + b K + c)* | Data points ≥ 4 | 48/49 | 47/49 | 3/98 |
| (*K, σ*) | Quadratic fit *(σ = a K^2^ + b K + c)* | a < 0 | 43/48 | 43/47 | 12/98 |

Table S3: *Invalid datasets for Popular stimuli analysis of primary data (N = 49)*

| RPT Graph | Fit type | Inclusion criteria | Approach curves | Avoidance curves | Total excluded curves |
| --- | --- | --- | --- | --- | --- |
| (*K*, *H*) | Boundary envelope | K > 0 at category level to produce H (Log of 0 is invalid) | 49/49 | 49/49 | 0/98 |
| (*K*, *H*) | Log fit (*H = a* log_10_*K + b*) | Data points ≥ 3 | 47/49 | 43/49 | 8/98 |
| (*K*, *H*) | Log fit (*H = a* log_10_*K + b*) | a > 0 | 47/47 | 43/43 | 8/98 |
| (*K*, *H*) | Power-law fit (*H = b K^a^*) | Data points ≥ 3 | 44/49 | 38/49 | 16/98 |
| (*K*, *H*) | Power-law fit (*H = b K^a^*) | 0 < a < 1 | 39/44 | 36/38 | 23/98 |
| (*K, σ*) | Boundary envelope | None | 49/49 | 49/49 | 49/49 |
| (*K, σ*) | Quadratic fit *(σ = a K^2^ + b K + c)* | Data points ≥ 4 | 47/49 | 43/49 | 8/98 |
| (*K, σ*) | Quadratic fit *(σ = a K^2^ + b K + c)* | a < 0 | 44/47 | 42/43 | 12/98 |

Table S4: *Invalid datasets for pooled Popular & Classical stimuli analysis of primary data (N = 49)*

| RPT Graph | Fit type | Inclusion criteria | Approach curves | Avoidance curves | Total excluded curves |
| --- | --- | --- | --- | --- | --- |
| (*K*, *H*) | Boundary envelope | K > 0 at category level to produce H (Log of 0 is invalid) | 49/49 | 49/49 | 0/98 |
| (*K*, *H*) | Log fit (*H = a* log_10_*K + b*) | Data points ≥ 3 | 49/49 | 49/49 | 0/98 |
| (*K*, *H*) | Log fit (*H = a* log_10_*K + b*) | a > 0 | 49/49 | 48/49 | 1/98 |
| (*K*, *H*) | Power-law fit (*H = b K^a^*) | Data points ≥ 3 | 48/49 | 47/49 | 3/98 |
| (*K*, *H*) | Power-law fit (*H = b K^a^*) | 0 < a < 1 | 46/48 | 45/47 | 7/98 |
| (*K, σ*) | Boundary envelope | None | 49/49 | 49/49 | 49/49 |
| (*K, σ*) | Quadratic fit *(σ = a K^2^ + b K + c)* | Data points ≥ 4 | 49/49 | 48/49 | 1/98 |
| (*K, σ*) | Quadratic fit *(σ = a K^2^ + b K + c)* | a < 0 | 47/49 | 48/48 | 3/98 |

Table S5: *Invalid datasets for the entire music RPT analysis (Summary)*

| Study | Stimuli | RPT Graph | Fit type | Inclusion criteria  for each case | Invalid datasets of approach | Invalid datasets of avoidance |
| --- | --- | --- | --- | --- | --- | --- |
| Pilot  (N=16) | Classical  Stimuli | (*K^±^*, *H^±^*) | Log fit  (*H = a* log_10_*K + b*) | 1. K > 0 | 0 | 0 |
|  |  |  |  | 2. Data points ≥ 3 | 0 | 0 |
|  |  |  |  | 3. *a* > 0 | 0 | 0 |
|  |  |  |  | **Subtotal of exclusion** | **0** | **0** |
|  |  |  | Power-law fit  (*H = b K^a^*) | 1. K > 0 | 0 | 0 |
|  |  |  |  | 2. Data points ≥ 3 | 1 | 1 |
|  |  |  |  | 3. 0 *< a <* 1 | 3 | 0 |
|  |  |  |  | **Subtotal of exclusion** | **4** | **1** |
|  |  | (*K^±^, σ^±^*) | Quadratic fit  *(σ = a K^2^ + b K + c)* | 1. Data points ≥ 4 | 0 | 0 |
|  |  |  |  | 2. *a* < 0 | 2 | 1 |
|  |  |  |  | **Subtotal of exclusion** | **2** | **1** |
|  | | | | | | |
| Primary  (N=49) | Classical Stimuli | (*K^±^*, *H^±^*) | Log fit  (*H = a* log_10_*K + b*) | 1. K > 0 | 0 | 0 |
|  |  |  |  | 2. Data points ≥ 3 | 1 | 2 |
|  |  |  |  | 3. *a* > 0 | 2 | 1 |
|  |  |  |  | **Subtotal of exclusion** | **3** | **3** |
|  |  |  | Power-law fit  (*H = b K^a^*) | 1. K > 0 | 0 | 0 |
|  |  |  |  | 2. Data points ≥ 3 | 9 | 4 |
|  |  |  |  | 3. 0 *< a <* 1 | 7 | 6 |
|  |  |  |  | **Subtotal of exclusion** | **16** | **10** |
|  |  | (*K^±^, σ ^±^*) | Quadratic fit  *(σ = a K^2^ + b K + c)* | 1. Data points ≥ 4 | 1 | 2 |
|  |  |  |  | 2. *a* < 0 | 5 | 4 |
|  |  |  |  | **Subtotal of exclusion** | **6** | **6** |
|  |  | | | | | |
|  | Popular  Stimuli | (*K^±^*, *H^±^*) | Log fit  (*H = a* log_10_*K + b*) | 1. K > 0 | 0 | 0 |
|  |  |  |  | 2. Data points ≥ 3 | 2 | 6 |
|  |  |  |  | 3. *a* > 0 | 0 | 0 |
|  |  |  |  | **Subtotal of exclusion** | **2** | **6** |
|  |  |  | Power-law fit  (*H = b K^a^*) | 1. K > 0 | 0 | 0 |
|  |  |  |  | 2. Data points ≥ 3 | 5 | 11 |
|  |  |  |  | 3. 0 *< a <* 1 | 5 | 2 |
|  |  |  |  | **Subtotal of exclusion** | **10** | **13** |
|  |  | (*K^±^, σ ^±^*) | Quadratic fit  *(σ = a K^2^ + b K + c)* | 1. Data points ≥ 4 | 2 | 6 |
|  |  |  |  | 2. *a* < 0 | 3 | 1 |
|  |  |  |  | **Subtotal of exclusion** | **5** | **7** |
|  |  | | | | | |
|  | Pooled  Classical and Popular Stimuli | (*K^±^*, *H^±^*) | Log fit  (*H = a* log_10_*K + b*) | 1. K > 0 | 0 | 0 |
|  |  |  |  | 2. Data points ≥ 3 | 0 | 1 |
|  |  |  |  | 3. *a* > 0 | 0 | 0 |
|  |  |  |  | **Subtotal of exclusion** | **0** | **1** |
|  |  |  | Power-law fit  (*H = b K^a^*) | 1. K > 0 | 0 | 0 |
|  |  |  |  | 2. Data points ≥ 3 | 1 | 2 |
|  |  |  |  | 3. 0 *< a <* 1 | 2 | 2 |
|  |  |  |  | **Subtotal of exclusion** | **3** | **4** |
|  |  | (*K^±^, σ ^±^*) | Quadratic fit  *(σ = a K^2^ + b K + c)* | 1. Data points ≥ 4 | 0 | 1 |
|  |  |  |  | 2. *a* < 0 | 2 | 0 |
|  |  |  |  | **Subtotal of exclusion** | **2** | **1** |

# Supplementary References

Benson, V., and Marano, M.A. (1998). Current estimates from the National Health Interview Survey, 1995. National Center for Health Statistics. *Vital Health Stat* 10(199).

Lecrubier, Y., Sheehan, D.V., Weiller, E., Amorim, P., Bonora, I., Harnett Sheehan, K., Janavs, J., Dunbar, G.C. (1997). The Mini International Neuropsychiatric Interview (MINI). A short diagnostic structured interview: reliability and validity according to the CIDI. *European Psychiatry,* 12(5), 224-231. ISSN 0924-9338, http://dx.doi.org/10.1016/S0924-9338(97)83296-8.

Oldfield, R. C. (1971). The assessment and analysis of handedness: the Edinburgh inventory. *Neuropsychologia, 9*(1), 97-113.

Wilkinson, G.S., & Robertson, G.J. (2006). *Wide Range Achievement Test: Fourth Edition.* Lutz, FL: Psychological Assessment Resources.
